# Hepatitis B virus persistence in mice reveals IL-21 and IL-33 as regulators of viral clearance

**DOI:** 10.1038/s41467-017-02304-7

**Published:** 2017-12-14

**Authors:** Zhongliang Shen, Huijuan Yang, Sisi Yang, Wei Wang, Xiaoxian Cui, Xian Zhou, Wei Liu, Shaokun Pan, Yanfeng Liu, Junqi Zhang, Jiming Zhang, Youhua Xie, Jing Liu

**Affiliations:** 1 Key Laboratory of Medical Molecular Virology (MOE/MOH) and Institutes of Biomedical Sciences, School of Basic Medical Sciences, Shanghai Medical College, Fudan University, 131 Dong’An Road, 200032 Shanghai, People’s Republic of China; 2 Department of Infectious Diseases, Huashan Hospital, Fudan University, 12 Middle Wulumuqi Road, 200040 Shanghai, People’s Republic of China; 3 Department of Surgery, Huashan Hospital, Fudan University, 12 Middle Wulumuqi Road, 200040 Shanghai, People’s Republic of China

## Abstract

Hepatitis B virus (HBV) generally causes self-limiting infection in immunocompetent adults, but establishes chronic infection in some adults and in most maternally infected infants. Factors determining clearance versus persistence are not fully understood. Hydrodynamic injection (HDI) of HBV replicon plasmid via tail vein generally results in quick clearance in immunocompetent adult mice. Here, we report the identification of strain-specific persistence of HBV in mice: one genotype B strain, designated BPS, persisted up to 33 weeks in ~50% of HDI mice. BPS persistence requires viral replication and multiple viral features. Compared to quickly cleared strains, BPS fails to induce robust post-exposure serum IL-21/IL-33 responses. Injection of IL-21-expressing or IL-33-expressing plasmids facilitates clearance of pre-established BPS persistence and protects cured mice from BPS re-challenge. IL-21 and IL-33 also induce clearance of pre-established HBV persistence in another mouse model. These data reveal IL-21 and IL-33 as potent regulators of HBV clearance and valid drug candidates.

## Abstract (web summary)

Hepatitis B virus (HBV) establishes chronic infection in only some patients, but the mechanisms underlying clearance failure in these patients are not fully understood. Here, the authors identify and characterize an HBV strain that can persist in mice and show that IL-21 and IL-33 responses contribute to clearance.

## Introduction

Hepatitis B virus (HBV) take humans as its sole natural host and exclusively infects hepatocytes in vivo^1^. Infection of naïve adults is usually asymptomatic or subclinical^2^, with 95% of symptomatic infections being self-limited, and the remaining 5% developing life-long chronicity. In contrast, nearly 90% of neonates and approximately 30% of children aged 1–5 years old progress to chronicity upon infection^3^. Chronic HBV infection is associated with higher risks of cirrhosis and hepatocellular carcinoma^4^. World Health Organisation estimated that HBV chronically infects ~240 million people worldwide and causes ~686,000 related deaths annually^5^.

Clinical indicators of recovery from acute hepatitis B, or failure of HBV to establish persistent infection, include loss of serum HBsAg and viral DNA, and appearance of HBsAb^2^. Host and/or viral factors determining clearance vs. persistence are not fully understood, partially due to scarcity of ideal animal models. Although chimpanzees develop chronic infection after experimental exposure^6^, routine use of this host is impractical. Mice are insusceptible to HBV infection, and transgenic mice expressing human HBV receptor NTCP^7^ apparently do not support infection either. However, transfected HBV genome is capable of expression and replication in mouse hepatocytes and murine models taking advantage of this fact are routinely used for characterising host-HBV interactions. For example, recent data linked age-dependent high chronicity rate in young mice to lower HBV-dependent IL-21 production^8,9^ and immature gut microbiota^10^. In comparison, such models have offered fairly limited information on HBV persistence in adults, mostly because adult mice of common immunocompetent strains, including BALB/c and C57BL/6, clear HBV serum markers very quickly after HBV genome plasmids were delivered via hydrodynamic injection (HDI)^11–13^. So far, relative persistence in naïve immunocompetent adult mice has only been observed when adeno-associated virus (AAV)^12^ or lentiviral^14^ sequences were included in delivery plasmid, or when low doses of recombinant adenovirus^15^ or AAV^16^ vectors were used for delivery. These models have enabled studying mechanisms of HBV persistence^17–19^ and developing novel therapeutics^20^, but it’s difficult to evaluate roles played by non-HBV viral elements in the observed effects, either directly or through interplay with HBV and/or host functions.

Here, we report the persistence of a clinical strain of genotype B HBV (designated BPS for ‘genotype B persistent strain’) in both BALB/c and C57BL/6 HDI mice without requiring non-HBV viral sequences. We characterised BPS-based HBV persistence mouse model by attempting to identify persistence-related viral and host factors, which showed IL-21 and IL-33 as potent inducers of HBV clearance and promising drug candidates.

## Results

### A clinical HBV isolate displays unique persistence in mice

**Fig. 1:**
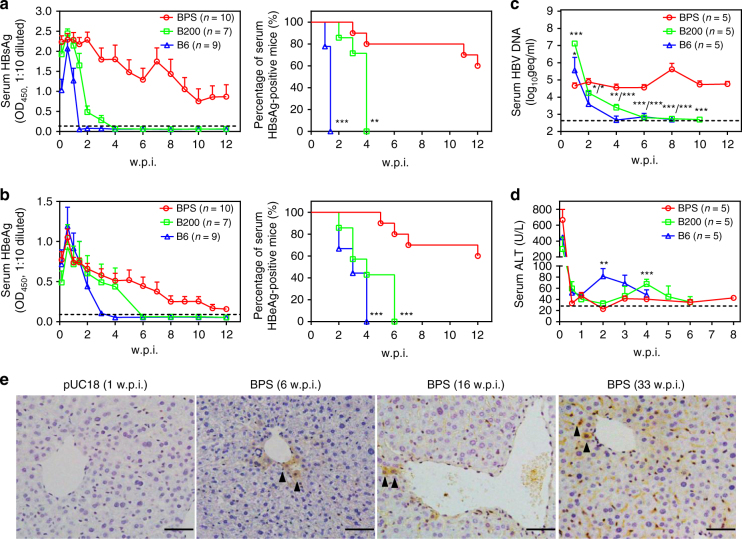
BPS persistence in BALB/c mice. Sera from BPS, B200 or B6 HDI mice were analysed for HBsAg (**a**), HBeAg (**b**), HBV DNA (**c**) and ALT (**d**). Group means and s.e.m. within group are presented with group sizes (*n*) indicated. Group positivity percentage data are also plotted for HBsAg (**a**, right panel) and HBeAg (**b**, right panel). Dotted lines represent cut-off thresholds (**a**, **b**, left panels), lower limit of quantification (**c**) or pre-injection baseline (**d**), respectively. B6 and B200 data are compared against BPS and statistical significance calculated using log-rank (Mantel–Cox) test (**a**, **b**, right panels) or unpaired two-tailed *t*-test (**c**, **d**). *p < 0.05; **p < 0.01; ***p < 0.001. **e** Liver sections taken from control and serum HBsAg positive BPS HDI mice were stained for HBsAg (arrow heads) in addition to H&E staining. Representative images from 2 mice are shown. w.p.i. weeks post injection. geq genome equivalents. Scale bars: 50 μm

To reproduce previous results regarding HBV HDI in immunocompetent adult mice, we hydrodynamically injected BALB/c mice with 1.3-fold over-length HBV genome on pUC18 backbone (Supplementary Table 1; Supplementary Fig. 1). Serum HBV antigens were measured in-house using ELISA and confirmed by commercial quantitative assay (Supplementary Fig. 2). As shown in Fig. 1, two genotype B clinical isolates (B6 and B200) were quickly cleared: serum HBsAg peaked at around 1 w.p.i. (weeks post injection), decreased thereafter and disappeared by 2 and 4 w.p.i. (Fig. 1a). Serum HBeAg followed a similar course and disappeared by 4 and 6 w.p.i. (Fig. 1b). Correspondingly, serum HBV DNA decreased from 1 w.p.i. onwards and became virtually undetectable by 8 w.p.i. (Fig. 1c). A panel of common non-B genotype strains also displayed similar quick clearance of HBsAg (Supplementary Fig. 3). These results agreed with previous data showing that HBV replicon plasmids without AAV-derived sequences could not persist in immunocompetent mice^12^.

One genotype B isolate designated BPS, however, displayed surprising persistence: serum antigens peaked at around 1 w.p.i. and started decreasing afterwards similarly to B6/B200, but persisted at considerable levels at 12 w.p.i. in 60% of BPS-injected mice (Fig. 1a, b). Serum HBV DNA also persisted at close to 1 w.p.i. levels (Fig. 1c). Furthermore, in repeated experiments with extended follow-up, serum HBV antigens and DNA remained positive up to 33 w.p.i. in half of BPS-injected mice, although antigen levels had decreased remarkably compared to 12 w.p.i. (Supplementary Fig. 4a–c). Immunohistochemistry also confirmed liver HBsAg expression in BPS HDI mice positive for serum HBsAg up to 33 w.p.i (Fig. 1e; Supplementary Fig. 4d).

All HDI mice displayed an acute, quickly resolved surge of serum ALT at day 1 p.i., most likely caused by HDI procedure. Afterwards, B6 and B200 HDI mice manifested ALT elevations at 2 and 4 w.p.i., respectively, which reverted to normal levels by 4 and 6 w.p.i. (Fig. 1d), roughly coinciding with disappearance of serum HBV antigens and DNA (Fig. 1a–c). In contrast, BPS HDI mice displayed only marginal ALT elevations following post-HDI ALT surge (Fig. 1d). Histochemistry performed on liver sections taken from HDI mice at 1 w.p.i. also showed marked immune infiltration in B6, but not BPS HDI mice, with similar HBV antigen staining in both (Supplementary Fig. 5). Such continued HBV expression and replication without sustained liver damage in BPS HDI mice is reminiscent of the inactive/immunotolerant chronic carrier state in human^2^. In mice harbouring BPS persistence, injected plasmid DNA in nuclei of liver cells serve as viral transcription templates without involvement of non-liver tissues, formation of cccDNA or chromosomal integration, which is identical to the situation in B6 HDI mice prior to clearance (Supplementary Fig. 6; Supplementary Table 2).

### Virological characterisation of BPS

**Fig. 2:**
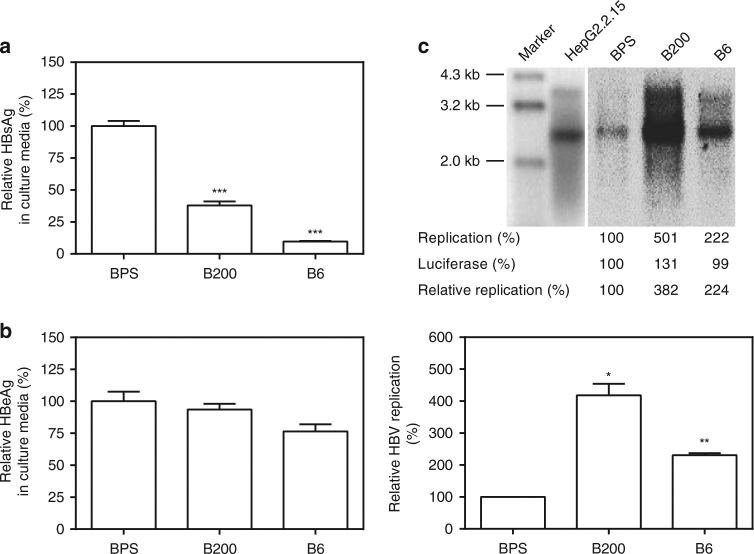
Comparison of viral expression and replication of BPS, B6 and B200 in vitro. HBV HDI plasmids were transfected into Huh-7 cells and culture supernatants at 48 h post transfection were assayed for HBsAg (**a**) and HBeAg (**b**) by ELISA. Intracellular capsid-associated HBV DNA was analysed in Southern blot (**c**, top panel), and quantified using densitometry scanning (**c**, bottom panel). HepG2.2.15 was analysed in parallel as signal position control. Transfection efficiency was normalised by measuring luciferase expressed from co-transfected pGL3 plasmid and data were normalised taking BPS measurements as 1. Transfections were performed in triplicates and repeated twice independently. Means and s.e.m. of the two repeats are plotted. **c** (top) shows representative Southern blot results. Statistical significance was calculated using unpaired two-tailed *t*-test (**a**, **b**, **c**, bottom panel). *p < 0.05; **p < 0.01; ***p < 0.001

Since similar persistence of BPS and quick clearance of B6/B200 were also observed in C57BL/6 mice (Supplementary Fig. 7), BPS persistence is most likely attributable to strain-specific viral features. Sequence alignment between BPS, B6 and B200 identified multiple amino acid differences in all ORFs, except preC, and nucleotide differences in all promoters and enhancers (Supplementary Tables 3, 4). BPS lacks preS2 start codon, but possesses no previously unreported variation. In transfected Huh-7 cells, BPS secreted more HBsAg, but similar HBeAg, compared to B6 and B200, and displayed lower replication (Fig. 2). Virions produced by BPS-transfected Huh-7 cells displayed no defect in infecting HepG2/NTCP cells (Supplementary Fig. 8). Clinical data on the origin patient of BPS showed no striking peculiarities either (Supplementary Table 1).

### BPS persistence requires multiple viral features

**Fig. 3:**
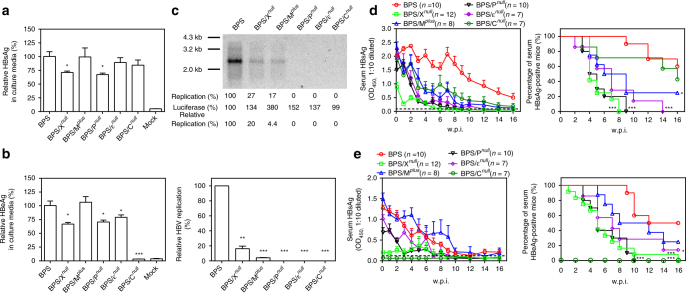
Comparison between BPS and BPS-derived mutants in vitro and in vivo. BPS and BPS-derived mutants with prematurely terminated C (C^*null*^), X (X^*null*^), or P (P^*null*^) ORFs, re-instated preS2 start codon (M^*plus*^) or inactivated packaging signal (ε^*null*^) were transfected into Huh-7 cells and supernatants were assayed for HBsAg (**a**) and HBeAg (**b**) at 48 hrs post transfection. **c** Intracellular capsid-associated HBV DNA was analysed in Southern blot, and quantified using densitometry scanning. Transfection efficiency was normalised by measuring luciferase expressed from co-transfected pGL3 plasmid and data were further normalised taking BPS measurements as 1. Transfections were performed in triplicates and repeated twice independently. Means and s.e.m. of the two repeats are plotted. **c** top panel shows representative Southern blot results. Plasmids were then used for HDI procedure on BALB/c mice. Sera were collected at indicated time points and analysed for HBsAg (**d**) and HBeAg (**e**). Group means and s.e.m. within group (left panels), as well as group positivity percentage data (right panels) are plotted with group sizes (*n*) indicated. Mutants’ data are compared against wild type and statistical significance calculated using unpaired two-tailed *t*-test (**a**, **b**, **c**, bottom panel) or log-rank (Mantel–Cox) test (**d**, **e**, right panels). *p < 0.05; **p < 0.01; ***p < 0.001. Dotted lines represent cut-off thresholds. w.p.i. weeks post injection

Roles of BPS features in persistence were then studied through targeted mutagenesis. Firstly, mutants with polymerase (BPS/P^*null*^), epsilon packaging signal (BPS/ε^*null*^), or HBcAg/HBeAg ORF (BPS/C^*null*^) obliterated were created (Supplementary Fig. 1), which displayed slightly decreased antigen secretion in transfected cells (Fig. 3a, b). Since polymerase, epsilon-mediated *cis*-packaging of pregenomic RNA by polymerase, and coating of pregenomic RNA/polymerase complex by HBcAg are essential for replication^1,2,21^, these mutants were expectedly replication-deficient (Fig. 3c). HDI of BPS/P^*null*^ and BPS/ε^*null*^ resulted in markedly lower levels of serum antigens, which disappeared in all BPS/P^*null*^-injected mice by 10 w.p.i., and 6 of 7 BPS/ε^*null*^-injected mice by 16 w.p.i. (Fig. 3d). BPS/C^*null*^-injected mice also manifested lower serum HBsAg, but clearance rate at 16 w.p.i. was comparable to BPS (Fig. 3d). However, 6 of 7 BPS/C^*null*^-injected mice cleared HBsAg by 20 w.p.i. (Supplementary Fig. 9). Clearly, BPS persistence requires viral replication and therefore is not a mere result of plasmid longevity. Slower clearance of BPS/C^*null*^ compared to other replication-deficient mutants suggests involvement of HBcAg/HBeAg in clearance, as previously reported using HBV plasmids containing AAV sequences^12,17^. Lowering injection doses has been linked with delayed clearance in certain HBV mouse models, suggesting effects of HBV replication level on host response^15^. However, clearance of neither B200 nor B6 was markedly affected when HDI dosage was lowered, and BPS at higher dosage still persisted in HDI mice (Supplementary Fig. 10).

Secondly, preS2 start codon was reinstated to create BPS/M^*plus*^ with mutation V1M(preS2)/R300H(P), identical to B6/B200 at this position (Supplementary Table 3). BPS/M^*plus*^ produced similar amounts of secreted antigens compared to BPS in vitro (Fig. 3a, b), and although BPS replicates more poorly than B6/B200 (Fig. 2c), the mutation making BPS/M^*plus*^ more B6/B200-like markedly decreased, rather than increased, replication competence (Fig. 3c). Nevertheless, in vitro infection of HepG2/NTCP cells by BPS/M^*plus*^ virions was comparable to wild-type BPS (Supplementary Fig. 8). BPS/M^*plus*^ HDI resulted in generally lower serum HBsAg than BPS (Fig. 3d), but comparable, sometimes higher, serum HBeAg (Fig. 3e). Overall clearance rate of serum HBsAg was also comparable to BPS/P^*null*^ and BPS/ε^*null*^, while clearance of serum HBeAg was slower than BPS/P^*null*^ and BPS/ε^*null*^, but still faster than BPS. Only about 20% of BPS/M^*plus*^-injected mice remained positive for serum antigens at 16 w.p.i. (Fig. 3e). Apparently, this notable variation only partially contributes toward persistence.

Thirdly, HBx ORF was obliterated to create BPS/X^*null*^, which displayed slightly decreased antigen expression, but markedly reduced replication (Fig. 3a–c) due to loss of HBx-mediated transactivation^22,23^. BPS/X^*null*^ HDI mice displayed markedly lower levels and quicker clearance of serum antigens compared to BPS and most other BPS-derived mutants, with HBsAg disappearing in all mice by 8 w.p.i. and HBeAg disappearing in all but one (*n* = 12) mice by 10 w.p.i. (Fig. 3d, e). Co-injection of BPS/X^*null*^ with BPS or B6 HBx resulted in markedly increased levels and extended presence of serum HBsAg and HBeAg, but by 16 w.p.i. these markers had dropped to detection limit levels in all co-injected mice (Supplementary Fig. 11). HBx is clearly necessary but not sufficient for full BPS persistence.

Finally, a series of chimeric constructs between BPS and B6 were constructed, but none of these displayed BPS-like persistence in HDI mice (Supplementary Figs. 12, 13). Taken together, these data demonstrated that replication and multiple viral features jointly contribute to persistence.

### BPS persistence correlates with failed immune response

**Fig. 4:**
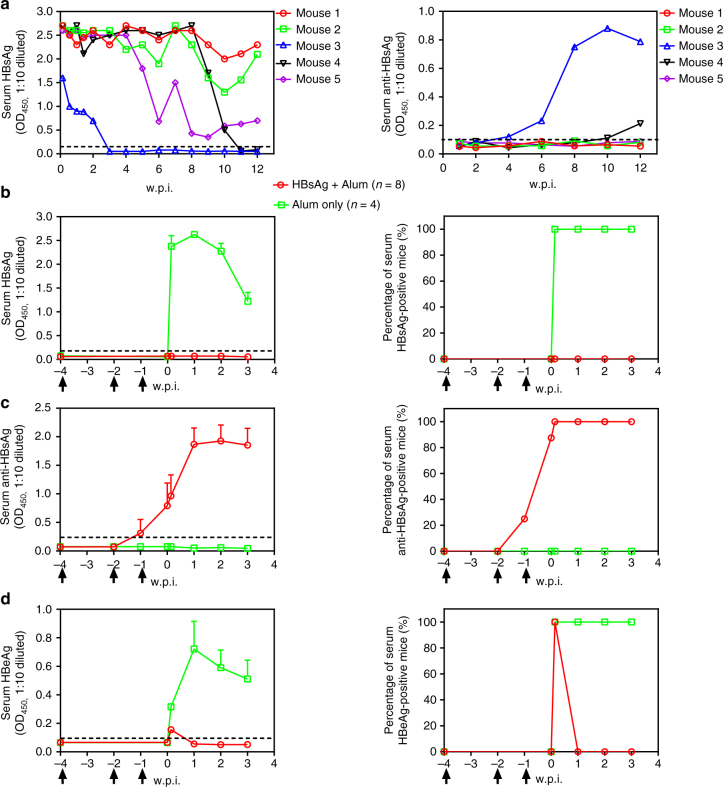
Relation between autonomous viral clearance in BPS HDI mice and serum HBsAb. **a** Sera from BPS HDI BALB/c mice were individually analysed for HBsAg (left panel) and HBsAb (right panel) using ELISA. Naive mice were then vaccinated with recombinant HBsAg in three doses at 4, 2 and 1 week(s) (arrows) prior to BPS HDI procedure. Sera were collected at indicated time points and analysed for HBsAg (**b**), HBsAb (**c**) and HBeAg (**d**) using ELISA. Group means and s.e.m. within group are presented with group sizes (*n*) indicated. Dotted lines represent cut-off thresholds. w.p.i. weeks post injection

Next, mouse responses to BPS vs. B6 and B200 were compared to identify host processes and factors affecting persistence vs. clearance. In athymic nude mice, BPS, B6 and B200 all persist for >6 w.p.i., demonstrating the necessity of immune competence for clearance (Supplementary Fig. 14). In immunocompetent mice, similar to previous reports^11,12^, correlation between HBsAb emergence and HBsAg clearance was clearly observed: HBsAb appeared quickly in B6-injected and B200-injected mice, roughly coinciding with HBsAg decrease and clearance (Supplementary Fig. 15), whereas 60% (*n* = 5) of BPS-injected mice failed to develop HBsAb by 12 w.p.i. and serum HBsAg persisted (Fig. 4), reproducing results in Fig. 1. The other two BPS HDI mice seroconverted to HBsAb at 4 and 10 w.p.i., respectively, with nearly concurrent disappearance of HBsAg (Fig. 4). For cellular responses, at 5 w.p.i., splenocytes from B6-injected mice contain more HBsAg-specific IFN-γ-producing cells than BPS-injected mice (Supplementary Fig. 16). Therefore, BPS persistence correlates with host’s failure to mount certain aspects of humoral and cellular responses. Accordingly, pre-exposure HBsAg vaccination induced HBsAb in all vaccinated mice and prevented BPS from establishing persistence (Fig. 4b, c). In contrast, HBsAg immunisation of mice with pre-established BPS persistence failed to induce HBsAb or clearance (Supplementary Fig. 17), indicating a state of immune tolerance not breakable by conventional vaccination, similar to human chronic infections^24^.

### IL-21 and IL-33 are associated with HBV clearance in mice

**Fig. 5:**
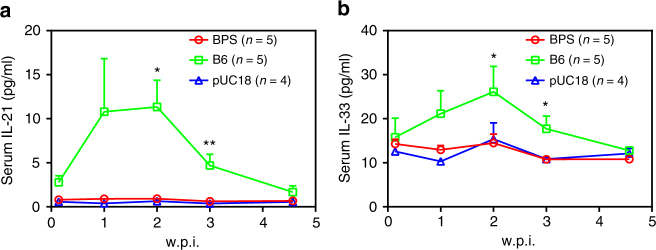
Serum IL-21 and IL-33 are stimulated in B6 HDI mice but not BPS HDI mice. Sera from BPS, B6 and pUC18 HDI BALB/c mice were collected at indicated time points and levels of selected cytokines quantitated using multiplex-based assay. Group means and s.e.m. within group of serum IL-21 (**a**) and IL-33 (**b**) measurements are presented with group sizes (*n*) indicated. BPS and B6 mice data are compared against pUC18 mice and statistical significance calculated using unpaired two-tailed *t*-test. *p < 0.05; **p < 0.01. For data on other tested cytokines, see Supplementary Fig. 18. w.p.i. weeks post injection

As cytokines play essential roles in both innate and adaptive immune responses, why mice responded differently to BPS was studied by comparing serum cytokine profiles of BPS-injected and B6-injected mice using Multiplex technology. Time points between 1 and 32 d.p.i. were selected, as serum HBV antigens have been cleared in B6-injected mice, but persist at considerable levels in BPS-injected mice at 4 w.p.i. (Fig. 1). TNF-α responses in vector-, BPS-injected and B6-injected mice were largely similar, whereas B6-injected mice displayed stronger IL-2, 4, 6 and 10 induction than BPS-injected mice (Supplementary Fig. 18). Most notably, IL-21 and IL-33 were not induced in vector-injected or BPS-injected mice, but strongly induced in B6-injected mice, peaking at 1–2 w.p.i. and reverting to normal levels by about 4 w.p.i. (Fig. 5). Post-exposure induction of IL-21 and IL-33 was also observed in B200, genotype A, as well as BPS/M^*plus*^ HDI mice (Supplementary Figs. 19, 20). Clearly, robust IL-21 and IL-33 responses to acute HBV exposure correlates with clearance in HDI mice. A similar association between IL-21 and clearance has been shown in another HBV mouse model^8^ and chronic human infections^25–27^.

### IL-21 and IL-33 functionally contribute towards clearance

To probe whether IL-21 and IL-33 actively participate in HBV clearance, B6-injected mice were subjected to repeated injections of anti-IL-21 or anti-IL-33 antibodies immediately. Mice treated with normal IgG controls cleared serum HBsAg and HBeAg by 2 and 4 w.p.i., similar to untreated mice (Fig. 1), whereas anti-IL-21 or anti-IL-33 injection notably alleviated the decrease and delayed the clearance of serum antigens by 2–4 weeks (Supplementary Fig. 21). Similar effects were observable when B6, B200 and genotype A HDI mice were subjected to repeated injections of combined anti-IL-21 and anti-IL-33 antibodies (Supplementary Figs. 22–24). Conversely, single injection with plasmids expressing mouse IL-21 or IL-33 (Supplementary Fig. 25) immediately after B6 or B200 HDI procedure slightly but noticeably facilitated decrease and clearance of serum HBV markers (Supplementary Figs. 26, 27). In contrast, single injection of IL-21 or IL-33 expression plasmids immediately after BPS HDI only marginally affected serum levels of HBsAg, HBeAg and HBV DNA, without markedly affecting persistence (Fig. 1; Supplementary Fig. 28). These data proved that IL-21 and IL-33, along with other factors, indeed actively contribute to HBV clearance in acute exposure.

### IL-21 and IL-33 induce clearance of BPS persistence

**Fig. 6:**
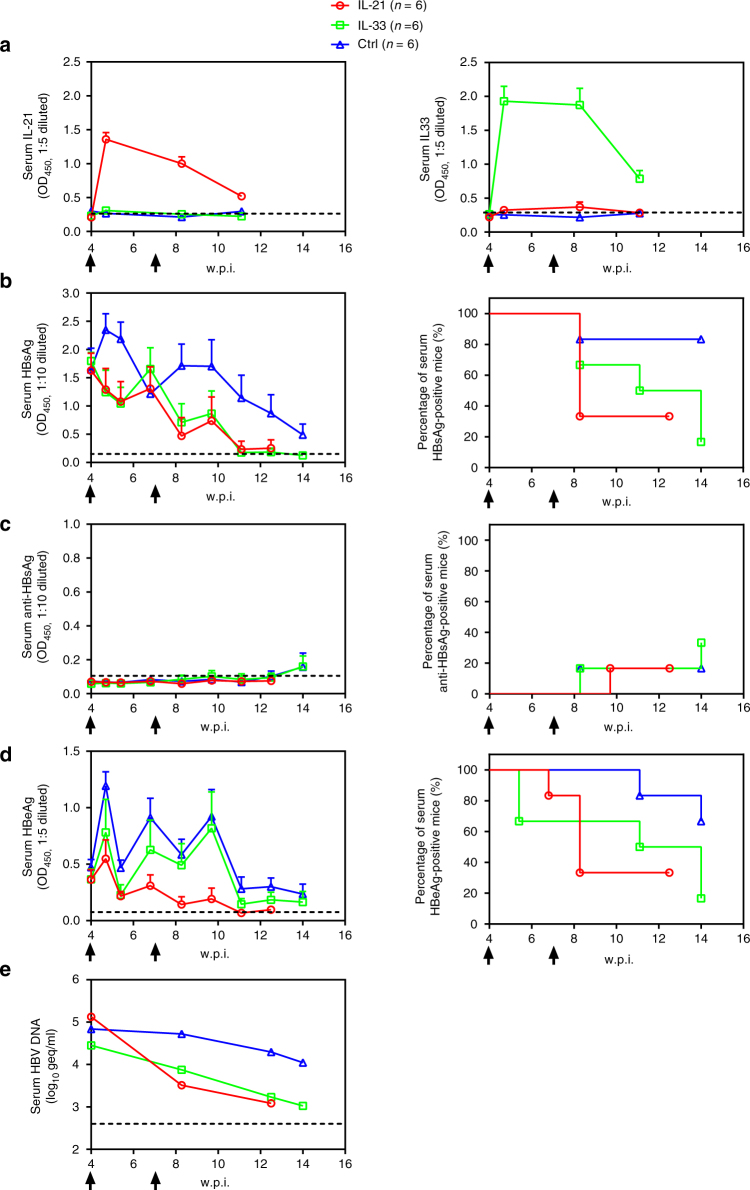
IL-21 or IL-33 treatments induce clearance of established BPS persistence. Plasmids expressing murine IL-21 or IL-33 and empty vector control were injected into BPS HDI mice at 4 and 7 weeks post injection (w.p.i.) (arrows). Sera were collected at indicated time points and levels of IL-21 and IL-33 (**a**), HBsAg (**b**), HBsAb (**c**) and HBeAg (**d**) were analysed using ELISA. Group means and s.e.m. within group are presented with group sizes (*n*) indicated. Group positivity percentage data are also presented for HBsAg (**b**, right panel), HBsAb (**c**, right panel) and HBeAg (**d**, right panel). HBV DNA for each group were analysed using pooled serum in commercial quantitative assay (**e**). Dotted lines represent cut-off thresholds (**a**, **b**–**d**, right panels) and lower limit of quantification (**e**) respectively. geq genome equivalents

We went on to test possible effects of IL-21 and IL-33 on established HBV persistence and hydrodynamically injected IL-21 or IL-33 expression plasmids into BPS HDI mice at 4 w.p.i., when BPS had established persistence while B6/B200 would have been cleared (Fig. 1), and once again at 7 w.p.i. Heightened serum IL-21 and IL-33 levels (Fig. 6a; Supplementary Fig. 29) correlated with decrease of serum HBV markers (Fig. 6b–d). More importantly, viral clearance rate was markedly improved: by around 8 w.p.i., 4 of 6 (67%) IL-21-treated mice had cleared serum HBV antigens, while 5 of 6 (83%) IL-33-injected mice cleared both HBV antigens by 14 w.p.i (Fig. 6b–d). Similarly, HBV DNA in pooled sera decreased rapidly following IL-21 or IL-33 treatments and dropped to detection limit levels by 14 w.p.i. (Fig. 6e). In contrast, 83 and 67% of mice injected with control plasmid failed to clear HBsAg and HBeAg by 14 w.p.i, respectively (Fig. 6b–d), comparable to untreated mice (Fig. 1). Combined injection of IL-21 and IL-33 plasmids displayed no significant additive effects compared to single injections (Supplementary Fig. 30). Treatment-induced disappearance of serum HBV markers was confirmed to be true clearance, instead of cytokine-induced repression of persisting BPS plasmid DNA (Supplementary Fig. 6), by analysing intrahepatic HBV DNA of responder and non-responder BPS mice (Supplementary Fig. 31). Histochemistry examination of liver sections showed more prominent immune infiltration in IL-21/IL-33 treated BPS HDI mice compared to mock treated controls and in responder mice, the infiltration apparently lasted longer (Supplementary Fig. 32). It appears that although exogenous IL-21 and IL-33 have limited repressive effects during acute phase of BPS exposure (Supplementary Fig. 28), treatments during chronic phase are highly effective and induce clearance in a majority of recipients.

Furthermore, IL-21 and IL-33 were tested on another mouse model of HBV persistence. C57BL/6 mice were injected via HDI with pAAV/HBV containing genotype A HBV genome and AAV-derived sequences^12^. At 4 w.p.i. ~ 60% injected mice failed to clear serum HBV antigens and these mice were injected with IL-21 and IL-33 plasmids in the same way as BPS HDI mice. Accelerated decrease and improved clearance of HBV markers, similar to but less markedly than in BPS mice, were observed (Supplementary Fig. 33). These data demonstrated that effects of IL-21 and IL-33 are not BPS, genotype B or BALB/c specific.

### IL-21-induced and IL-33-induced clearance confers protection

**Fig. 7:**
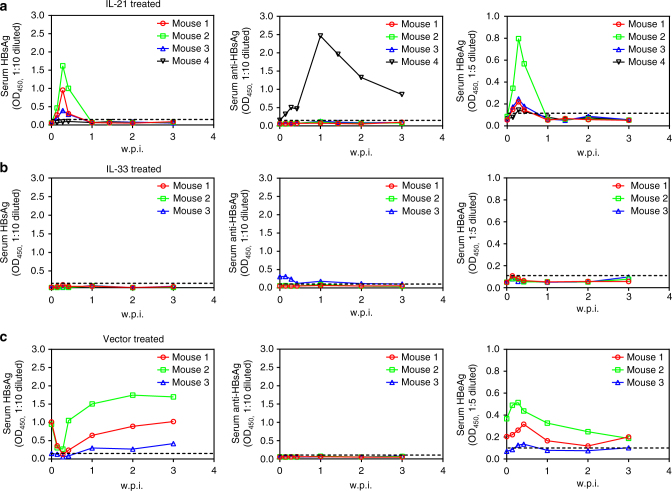
IL-21-induced or IL-33-induced clearance of BPS persistence engenders protection from re-challenge. HDI mice harbouring BPS persistence were treated with injections of plasmids expressing IL-21 (**a**) or IL-33 (**b**) at 4 and 7 w.p.i. and those displaying at least 4 weeks of sustained HBsAg negativity (see Fig. 6) were re-challenged in the same way as initial BPS HDI. BPS HDI mice treated with vector control were used as control and re-challenged similarly (**c**). Sera were collected at indicated time points and levels of HBsAg (left), HBsAb (middle) and HBeAg (right) were analysed using ELISA. Dotted lines represent cut-off thresholds. w.p.i. weeks post injection of BPS re-challenge

Autonomous clearance of HBV in HDI mice correlates with HBsAb seroconversion (Fig. 4; Supplementary Fig. 15). In IL-21 and IL-33 treated BPS HDI mice that cleared HBV markers, however, only 1 of 4 and 2 of 5 mice developed HBsAb, respectively (Fig. 6c), indicating the effects of IL-21 and IL-33 on BPS are HBsAb-independent. More surprisingly, when mice that cleared BPS in response to IL-21 or IL-33 were re-challenged with BPS, no persistence was observed: all mice cleared HBsAg and HBeAg within a week (Fig. 7). Once again, there was no marked seroconversion to HBsAb: only 1 of 4 IL-21-cured and 1 of 3 IL-33-cured mice developed HBsAb after re-challenge (Fig. 7a, b). Apparently IL-21 and IL-33 induce a lasting HBsAb-independent functional resistance to HBV, suggesting new prophylactic and therapeutic possibilities.

## Discussion

HBV does not infect mouse hepatocytes and consequently, transfection-based mouse models of HBV, including HDI mice, do not reflect full viral life cycle. Nevertheless, these models enable analysis and comparison of virus-host interactions in mice and human. Unlike previously reported mouse models of HBV persistence, BPS persists in common immunocompetent mice without requiring non-HBV viral elements. Persistence is highly strain-specific (Fig. 1; Supplementary Fig. 3), whereas no strain-outcome association has been confirmed in human HBV infections. Human genetic heterogeneity might be a factor at work here, but BPS can persist in at least two diverse mouse strains: BALB/c (H-2^d^) and C57BL/6 (H-2^b^) (Fig. 1; Supplementary Fig. 7). Conceivably, capability of most, if not all, HBV strains to establish chronicity in human reflects evolutionary adaptation to its natural host. In this sense, BPS represents an exception compared to the majority of HBV strains that the non-natural host, namely mouse, quickly clears. Identification of such an exception offers a unique opportunity for studying mechanisms mediating HBV clearance and persistence in a tractable in vivo system, with no interference from non-HBV viral sequences.

Strain-specific persistence indicates involvement of viral factors in determining outcome in HDI mice. BPS harbours no novel variation, and targeted mutations and segment swapping showed that multiple BPS features simultaneously contribute towards persistence (Fig. 3; Supplementary Figs. 12, 13). Other HBV strains with combined features enabling BPS-like persistence in HDI mice could exist, but previous data and our results suggest that such strains could be rare.

At functional level, BPS displayed higher HBsAg expression and lower replication in transfected cells (Fig. 2), but in HDI mice, serum HBsAg levels during acute phase (0-2 w.p.i.) were comparable between BPS and B6/B200 (Fig. 1a), while serum HBV DNA levels correlated with replication competences in vitro, with BPS being the lowest (Figs. 1c, 2c). Whether lower replication plays any part in BPS persistence is uncertain, but our data suggested that low(er) replication alone is not a decisive factor: BPS-derived mutants BPS/X^*null*^ and BPS/M^*plus*^ displayed decreased replication (Fig. 3c), but were cleared more quickly (Fig. 3d, e), and BPS-B6 chimeras displaying lower-than-B6, BPS-like replication competence were all cleared within 8 w.p.i. (Supplementary Figs. 12, 13). Furthermore, BPS at increased or reduced HDI dosage still causes persistence (Supplementary Fig. 10).

Interestingly, despite its comparatively lower replication competence, BPS requires replication to persist in HDI mice (Fig. 3). As HBV does not infect mouse hepatocytes, replication could not promote persistence through expanding infection. Intracellular BPS replication and exported mature virions may directly or indirectly hinder host responses that clear transfected hepatocytes. Since such processes are not unique to BPS, the singular persistence of BPS suggests that persistence-related BPS features discussed above are more important.

Viral factors need to work through affecting host responses to engender persistence, as reflected in the facts that BPS, B6 and B200 all persist in athymic nude mice (Supplementary Fig. 14), whereas BPS is quickly cleared in HBsAg-vaccinated immunocompetent mice (Fig. 4). Humoral and cellular responses to BPS vs. B6 are expectedly different (Fig. 4; Supplementary Fig. 16), but more note-worthy differences were observed in cytokine profiles (Fig. 5; Supplementary Fig. 18). Identification of IL-21 and IL-33 as under-stimulated in BPS HDI mice (Fig. 5; Supplementary Figs. 19, 20) echoes previous observations implicating IL-21 in HBV persistence in young mice^8^ and human adults^25–27^. Mechanistic involvement of IL-21 and IL-33 in HBV clearance is supported by effects of administrating antibodies or expression plasmid during acute phase in HDI mice injected with B6 or other quickly cleared strains (Supplementary Figs. 21–24, 26, 27), and most interestingly, by IL-21-induced and IL-33-induced clearance of pre-established BPS persistence (Fig. 6). Elucidating the origins and targets of IL-21 and IL-33, BPS-specific features preventing or counteracting their induction, and the underlying mechanisms warrants immediate efforts. Since there is no cross-induction between IL-21 and IL-33 (Fig. 6a), and no significant additive or synergistic effects were observable when both interleukins were blocked or administered (Supplementary Figs. 21, 22, 30), it’s likely that these factors independently act through similar downstream processes to induce clearance. Our preliminary results showed that treatment with these cytokines induced immune infiltration in liver of mice harbouring BPS persistence and prolonged infiltration is apparently associated with eventual clearance (Supplementary Fig. 32). Admittedly, cytokines untested here, including but not limited to those shown in Supplementary Figs. 16, 18, may also be important either individually or collectively, but IL-21-induced and IL-33-induced clearance of BPS persistence suggested that these two alone can cause pivotal changes in host responses. Similar effects of IL-21 and IL-33 in another HBV persistence mouse model (Supplementary Fig. 33) reconfirmed them as potent regulators of HBV clearance.

HBsAb seroconversion is a recognised hallmark of recovery and protection in both human and animals^2^. The finding that IL-21-induced or IL-33-induced BPS clearance is HBsAb-independent (Fig. 6), but engenders lasting protection (Fig. 7) is both surprising and promising. Understanding the cellular and molecular nature of such clearance and protection may reveal new aspects of anti-HBV host responses with prophylactic and therapeutic relevance. Meanwhile, these data support IL-21 and IL-33 as valid drug candidates for immunotherapy of chronic HBV infections. These can be used, after taking into consideration potential immune-related side-effects and establishment of corresponding counter-measures, either alone or in combination with antiviral chemotherapy that reduces viral load, as in BPS model IL-21 or IL-33 administration during persistence with reduced viral activity was more effective compared to administration during acute phase (Fig. 6 and Supplementary Fig. 28). On the other hand, it is also possible that a more optimised administration scheme, which could additionally involve other potentially clearance-related immune regulator(s) as discussed above, might enable IL-21 or IL-33 to produce improved results in acute exposure as well.

## Methods

### HBV plasmids and antibodies

GenBank Accession Numbers of HBV strains used and clinical data on origin of patients are listed in Supplementary Table 1. BPS was previously reported^28^. B200 and B6 were isolated from patients of Huashan Hospital with informed consent obtained in compliance with requirements of Huashan Hospital Bioethics Committee (Permit No. KY2014-024). Genotype D strain was a gift from Dr. Yongxiang Wang, Fudan University, and other strains were chemically synthesised. HDI plasmids were constructed by cloning ~1.3 fold over-length HBV genome into pUC18 (Takara, China) (Supplementary Fig. 1a). BPS/X^*null*^, BPS/P^*null*^ and BPS/C^*null*^ were created by prematurely terminating X, P and C ORFs (Supplementary Fig. 1b, d). BPS/ε^*null*^ was created by introducing inactivating mutations^21^ in ε packaging signal (Supplementary Fig. 1c, d). Mutations were designed to change overlapping ORF(s) only synonymously. BPS/M^*plus*^ was created by introducing V1M mutation into preS2 ORF, with concurrent R300H mutation in overlapping P ORF (Supplementary Fig. 1b, d). HBx of BPS and B6 were cloned downstream of CMV promoter and FLAG tag in pCMV-N-Flag vector (Beyotime, China) to create pBPSX and pB6X. X^*null*^ mutation was introduced into pB6X to create pB6Xm. Luciferase-expressing pGL3 (Promega, China) was used as transfection control. HBV HDI plasmid harbouring AAV sequences (pAAV/HBV) was a gift from Professor Pei-Jer Chen, National Taiwan University^12^. Mouse IL-21 (MG50137-M-N) and IL-33 (MG50118-M-N) expression plasmids (Sino Biological, China) and rat monoclonal antibodies (anti-mIL-21: 16-9333; anti-mIL-33, 16-7211) (eBioscience, China) were purchased.

### Transfection and analysis of HBV markers and virions

Culture and transfection of Huh-7 (TCHu182, Cell Bank of Chinese Academy of Sciences, China) and HepG2 (American Tissue Culture Collection, ATCC^®^ Number: HB-8065™, Manassas, VA) cells were performed as previously described^22,29^. Secreted HBV antigens were measured by ELISA (KHB, China) at 48 h post-transfection. Intracellular proteins were analysed using harvested cells in Western blot and antibodies against HBsAg (1:3000, NB100-62652, Novus), FLAG (1:3000, F3165, Sigma) and actin (1:50,000, A3584, Sigma). Original uncropped Western blots are shown in Supplementary Fig. 34. Intracellular capsid-associated HBV DNA was extracted and analysed in Southern blot using digoxigenin-labelled probes as previously described^22,29^. Briefly, transfected cells were lysed in 50 mM Tris/HCl (pH 7.5) containing 1% NP-40 and 8% sucrose at 37 °C for 15 min. DNase I (Sigma) and RNase A (Sigma) were then added to a final concentration of 100 μg/ml plus 10 mM MgCl_2_ to degrade free nucleic acids at 37 °C for 1 h. Lysates were cleared by centrifugation and treated with 100 μg/ml proteinase K (Merck) in 25 mM EDTA, 150 mM NaCl and 1% SDS at 37 °C overnight. Viral DNA was finally recovered by phenol/chloroform extraction and ethanol precipitation, and separated on a 1% agarose gel. After denaturation in 400 mM NaOH and 3 M NaCl, DNA was transferred onto nylon membrane (GE Amersham), hybridised with digoxigenin-labelled HBV-specific probe encompassing nucleotides 1901-3215/1-1185 of BPS genome prepared using PCR DIG labelling kit (Roche), and detected using DIG Luminescent Detection Kit (Roche). Original uncropped Southern blots are shown in Supplementary Fig. 35. Transfection experiments were repeated at least twice. For HBV infection assay, virions in transfection supernatants were concentrated by ultrafiltration in Amicon Ultra-15 tubes with molecular weight cut-off of 100 KDa and about 1 × 10^7^ HBV genome equivalents (geq), as determined using real-time PCR HBV DNA detection kit (Qiagen), were used for infecting 1 × 10^5^ HepG2 cells stably transfected with human NTCP (HepG2/NTCP) in the presence of 4% polyethylene glycol 8000 (Sigma) and 2.5% dimethyl sulfoxide (DMSO) (Sigma) as previously reported^7^. Eight hours post infection, cells were washed with PBS and changed into fresh media with subsequent media changes every 1–2 days.

### Mice work

Male 6-week-old to 8-week-old specific pathogen-free BALB/c and C57BL/6 mice were purchased from Experimental Animal Centre, Fudan University. Group sizes were chosen generally based on Mead’s resource equation. No blinding or special randomisation was used. For HDI, 10 μg plasmid DNA was injected into the tail vein in a volume of PBS equivalent to 8% of body weight delivered within 5–6 seconds. For HBx *trans*-complementation, 10 μg BPS/X^null^ was co-injected with 10 μg HBx expression plasmid. HBsAg vaccination was performed by injecting mice with 4 μg of yeast-expressed HBsAg (kindly provided by Professor Di Qu, Fudan University) emulsified in 0.1 ml PBS containing 0.5 mg Al(OH)_3_ (Sigma) into caudal thigh muscles. For antibody treatment, mice were injected intraperitoneally with 10 μg anti-IL-21, anti-IL-33 or normal IgG at 2–3 day intervals starting at 4–8 h post HDI. For interleukin treatment, mice were injected through HDI with 25 μg plasmid expressing murine IL-21 or IL-33. IL-treated BPS HDI mice displaying >4 week HBsAg negativity were re-challenged as initial BPS HDI. For pAAV/HBV model, C57BL/6 mice were injected via HDI with 10 μg pAAV/HBV and at 4 w.p.i., mice with positive serum HBV antigens were selected and injected with interleukin plasmids as above. Sera were collected through retro-orbital sinus bleeding, and tissues were collected after sacrifice by cervical dislocation. Mouse procedures were approved by the Animal Ethics Committee of School of Basic Medical Sciences, Fudan University.

### Serum analyses

Serum HBsAg, HBeAg and HBsAb levels were analysed using ELISA (KHB, China), initially validated by commercial quantitative assays (Adicon, China) performed in parallel (Supplementary Fig. 2). Serum HBV DNA and ALT were analysed using commercial quantitative assays (Adicon, China). Cytokine profiling was performed using Milliplex multiplex assay (Merck Millipore, China) on a Luminex 200 (Luminex, China). IL-21 and IL-33 in culture media and serum were detected using ELISA (Boster, China).

### ELISPOT and immunohistochemistry

Splenocytes cultured in RPMI 1640 (Invitrogen) containing 2 mM L-glutamine, 50 U/ml penicillin, and 10% foetal bovine serum (Invitrogen) were stimulated with 15 μg/ml recombinant HBsAg for 18 h. Gamma interferon (IFN-γ)-secreting cells were detected using IFN-γ ELISPOT assay (eBioscience, China) and spots were analysed using an iSpot Reader (AID, Germany). For immunochemistry, liver tissue sections were fixed and stained using anti-HBsAg (1:2, 000, R-0283-02, ChangDao, China) or anti-HBcAg (1:2, 000, GB058601, GeneTech, China) antibodies in addition to hematoxylin and eosin (H&E) staining.

### Analysis of HBV plasmid DNA in mouse tissue

To extract total nuclear DNA, HDI mouse tissues were lysed using TBS (10 mM Tris-HCl at pH 7.0, 150 mM NaCl) containing 0.5% Nonidet P-40. Released nuclei were washed 5 times with TBS and pelleted by brief centrifugation. Pelleted nuclei were subjected to lysis in 25 mM Tris-HCl at pH 8.0 with 25 mM EDTA, 125 mM NaCl, 1 mg/ml protease K and 1% SDS. After incubation at 55 °C overnight, released nuclear DNA was further extracted with phenol and chloroform, followed by precipitation using ethanol. Extracted nuclear DNA was then analysed for the presence of HBV and/or injected plasmid DNA in PCR or quantitative real-time PCR using mouse *GAPDH* gene as internal control. HBV transgenic mouse (a gift from Prof. Qiang Deng of Fudan University) was used as positive control. Sequences of primers used are listed in Supplementary Table 2. Form of HBV-related DNA was analysed in Southern blot as above with or without prior restriction enzyme treatment. Original uncropped agarose gel images and Southern blots are shown in Supplementary Fig. 36.

### Data and statistical analysis

ELISA and scanned Southern blot data were first normalised for transfection variation using luciferase readings and then normalised taking BPS data as 1 (100%). Means and standard errors (s.e.m.) from independently repeated experiments are plotted and subjected to unpaired two-tailed *t*-test. Mouse data are presented as group means and s.e.m. or for each mouse individually as indicated. Group positivity percentages are presented as Kaplan-Meier plot and subjected to log-rank (Mantel–Cox) test when necessary. GraphPad 5 was used for plotting and statistical tests.

### Data availability

Relevant sequence data referenced in this study are listed in Supplementary Table 1. All other data that support the findings of this study are available from the corresponding authors upon request.

## Electronic supplementary material

Supplementary Information
