# Supplementary material for: Hepatitis B virus persistence in mice reveals IL-21 and IL-33 as regulators of viral clearance
Source: Nat Commun. 2017 Dec 14;8:2119. doi: 10.1038/s41467-017-02304-7 (PMC5730569; doi:10.1038/s41467-017-02304-7)
Supplement: Supplementary file 1 — Supplementary Information [file 41467_2017_2304_MOESM1_ESM.pdf]

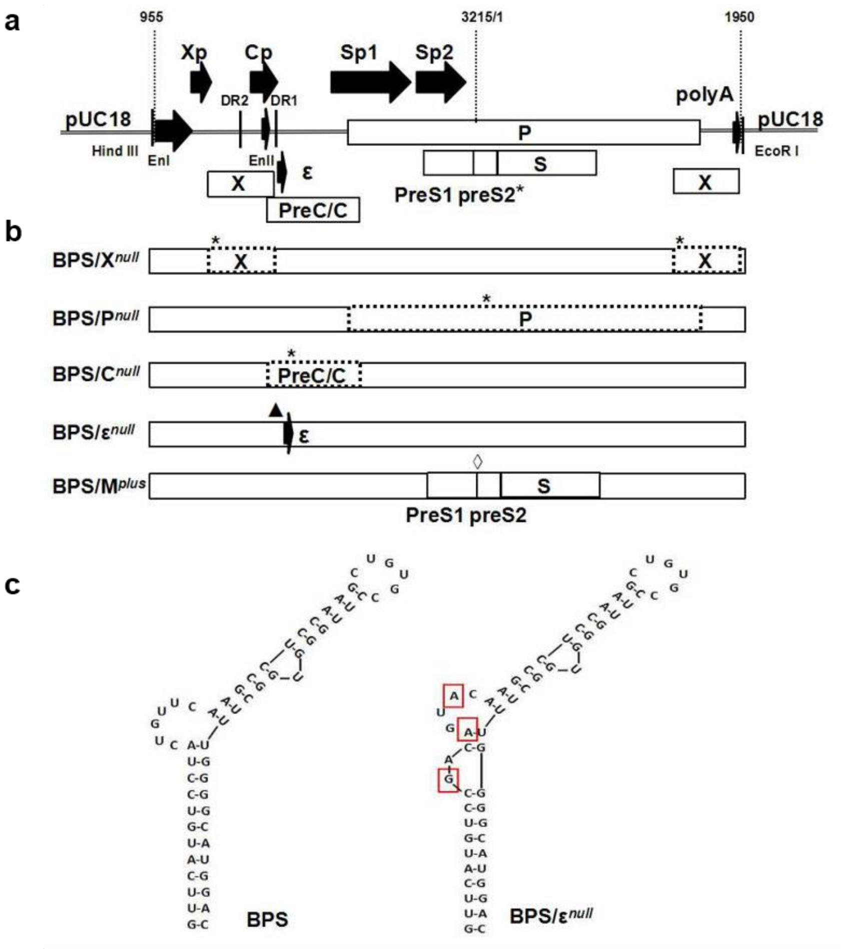

d

Mutants	Nucleotide	ORF
BPS/X^{null}	C1395T	X:Q8ter
BPS/P^{null}	G151T	P:E354ter
BPS/C^{null}	A2096T/T2097A	C:M66ter
BPS/M^{plus}	G3205A	preS2: V1M, P: R300H
BPS/ε^{null}	T1858G/T1861A/T1864A	/

Supplementary Figure 1 BPS plasmid and BPS-derived mutants used for HDI. **a** Schematic representation of BPS replicon plasmid used for HDI. Terminally redundant (~1.3 fold) HBV genome was inserted into pUC18 using the restriction enzyme sites indicated. Start and end of HBV sequences and their nucleotide positions on HBV genome are indicated. ORFs, promoters/enhancers, direct repeats DR1 and DR2, epsilon packaging signal (ε) and polyA signal are also indicated. Other HBV strains used in this work were cloned in a similar fashion. *, BPS lacks preS2 start codon and has valine instead of methionine at the position. **b** Schematic representation of BPS-derived mutants used in this work. Only elements affected by site-directed mutations are shown and obliterated ORFs are highlighted with dashed lines. *, artificially introduced terminator codon. ▲, ε-inactivating mutations. ◊, artificially introduced start codon. **c** Secondary structure modelling of ε packaging signal of BPS and BPS/ε^{null} using RNAfold (http://rna.tbi.univie.ac.at/cgi-bin/RNAfold.cgi). Nucleotides affected by the mutation are highlighted. **d** Nucleotide and amino acid changes in BPS-derived mutants.

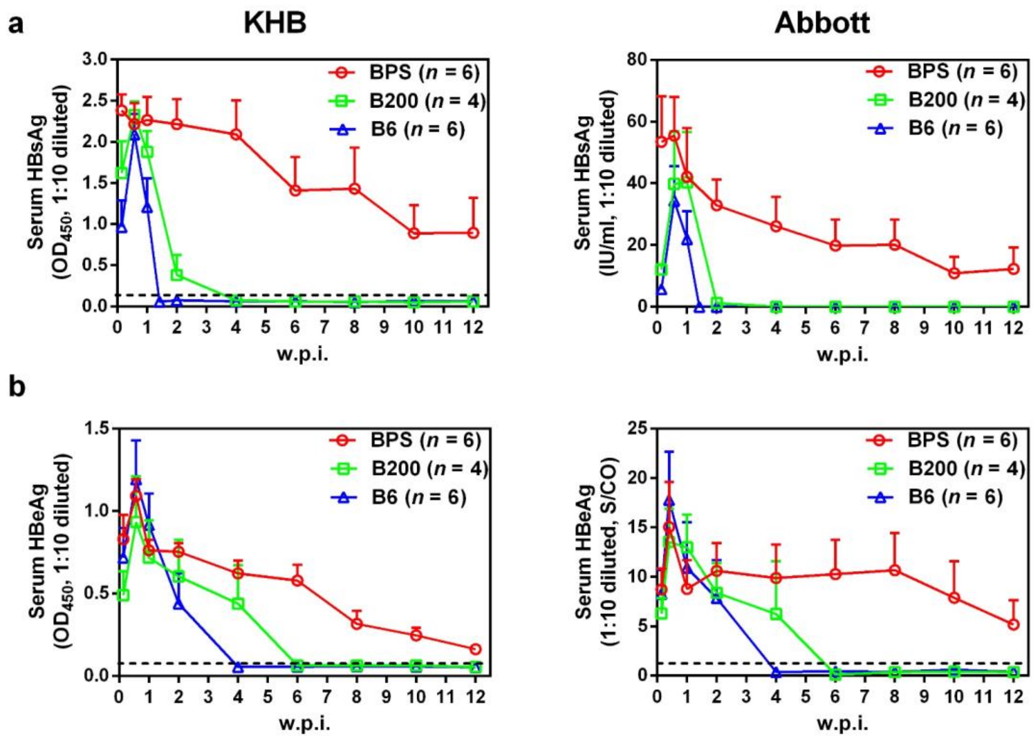

Supplementary Figure 2 Validation of ELISA results using commercial quantitative assays. A single dose of 10 μg HBV replicon plasmid containing 1.3 fold overlength genome of BPS, B200 or B6 was injected via HDI into 6-8 weeks old male BALB/c mice. Sera were collected at indicated time points and analysed for HBsAg **(a)** and HBeAg **(b)** using both KHB ELISA performed in our lab (left) and commercial Abbott quantitative assays (right). Group means and s.e.m. within group are presented with group sizes (*n*) indicated. Dotted lines represent cut-off thresholds (**a&b,** left & **b**, right). w.p.i., weeks post injection.

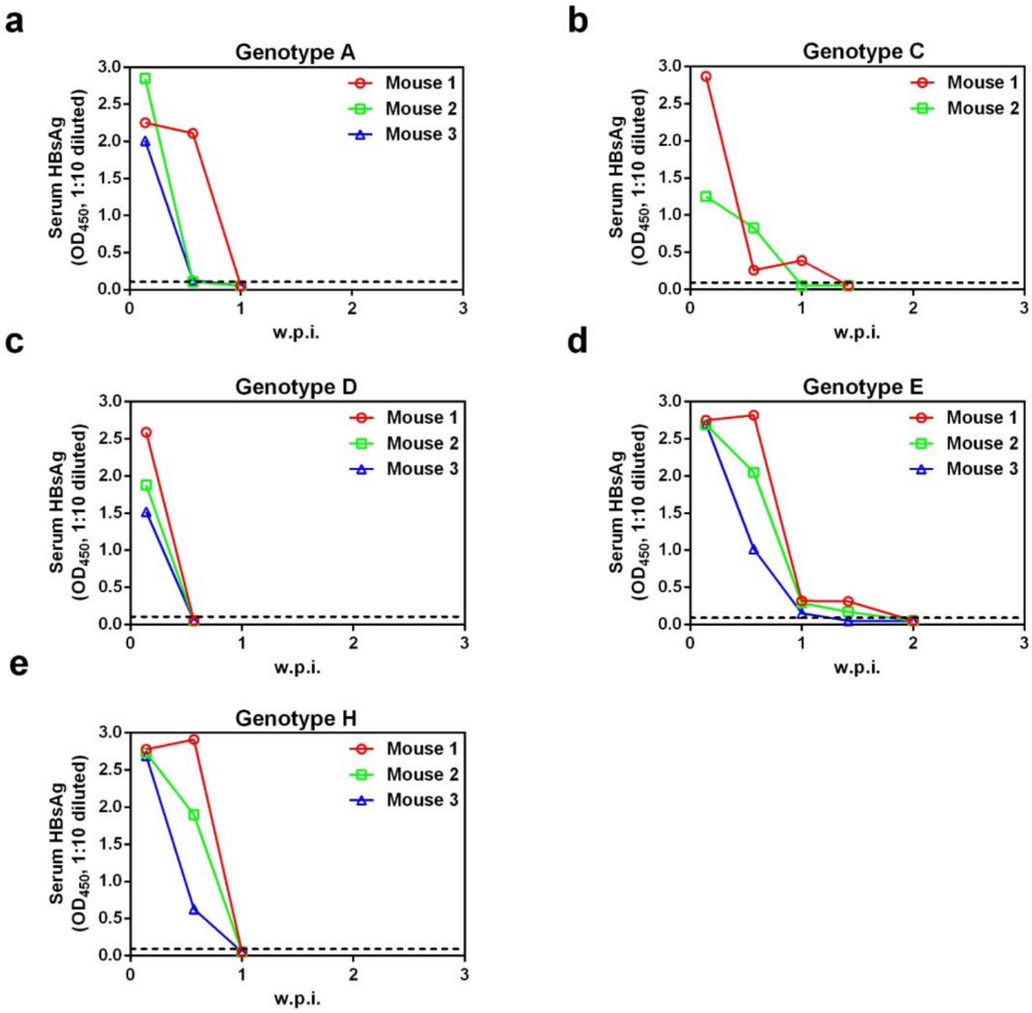

Supplementary Figure 3 Outcome of Non-B genotypes of HBV after HDI in BALB/c mice. HBV replicon plasmids derived from indicated genotypes (details listed in Supplementary Table 1) were injected into 6-8 weeks old male BALB/c mice via HDI. Sera were collected at indicated time points and analysed for HBsAg using ELISA. Dotted lines represent cut-off thresholds. w.p.i., weeks post injection.

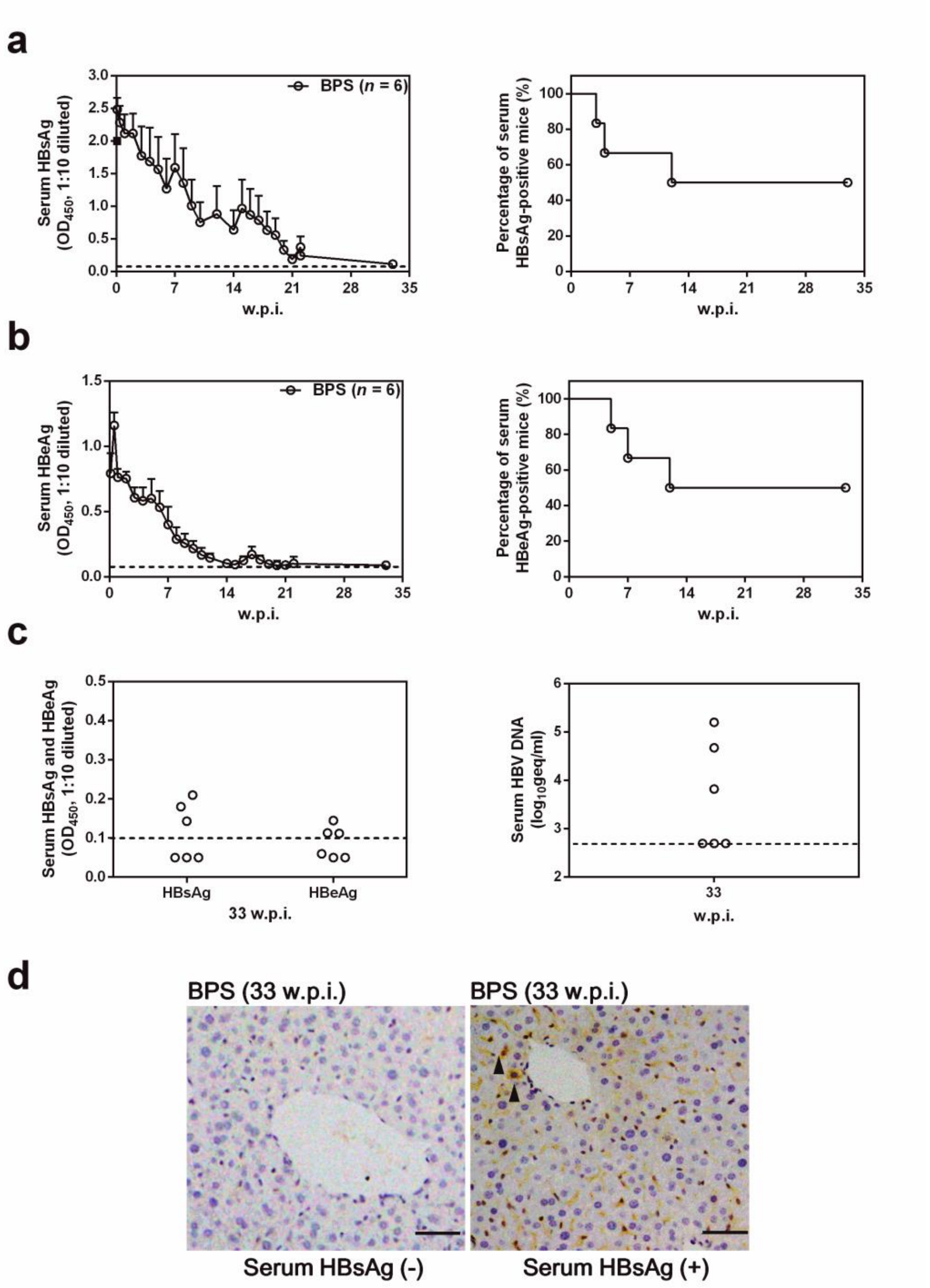

Supplementary Figure 4 Extended follow-up of BPS HDI mice. A single dose of 10 μg BPS replicon plasmid was injected into 6-8 weeks old male BALB/c mice (*n* = 6) via HDI. Sera were collected at indicated time points and analysed for HBsAg **(a)** and HBeAg **(b)** using ELISA. Group means and s.e.m. within group (left) and group positivity percentage data (right) are presented. **c** Serum HBV antigen (left) and DNA (right) levels at 33 w.p.i. were analysed using ELISA and commercial quantitative assay respectively. Dotted lines represent cut-off thresholds (**a&b**) and lower limit of quantification (**c**) respectively. w.p.i., weeks post injection. geq, genome equivalents. **d** Liver sections taken from serum HBsAg negative and positive BPS HDI mice at 33 w.p.i. were stained for HBsAg (arrows) in addition to H&E staining. Representative images from 2 mice in each group are shown. Scale bars: 50 μm.

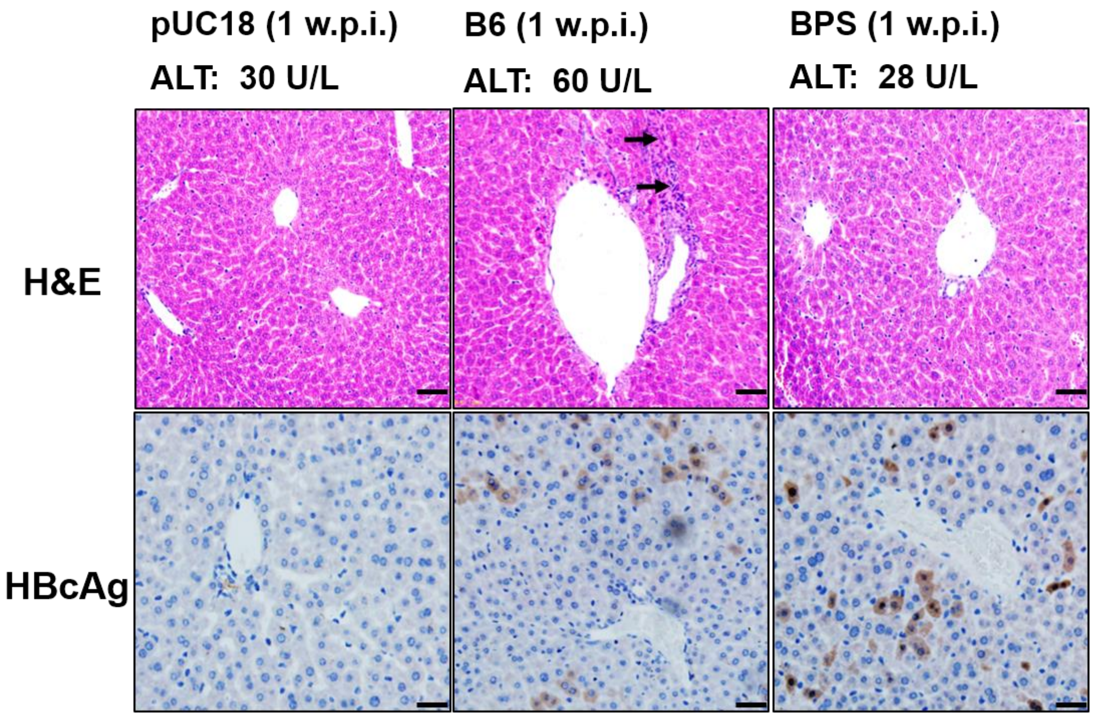

Supplementary Figure 5 Immune infiltration in liver of post HDI mice during acute phase. A single dose of 10 μg HBV replicon plasmid or empty vector control as indicated was injected into 6-8 weeks old male BALB/c mice via HDI. Liver sections were taken at 1 w.p.i. and subjected to H&E staining (top) and additional HBcAg immunostaining (bottom). Representative images from each group (n = 3) are shown along with ALT measurements from the corresponding mice. Arrows: infiltration foci. Scale bars: 50 μm. w.p.i., weeks post injection.

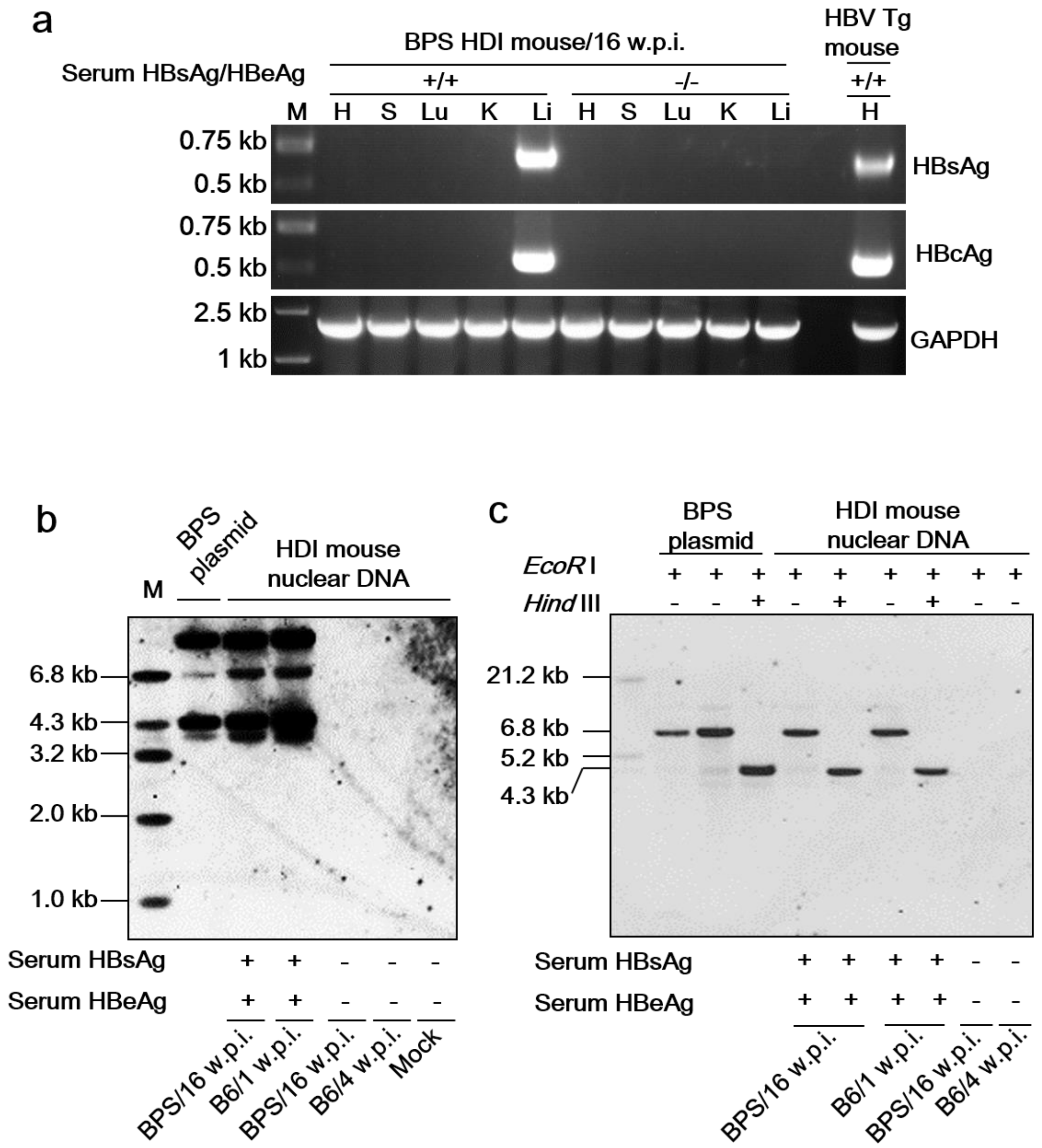

Supplementary Figure 6 Characterization of HBV transcription template in HDI mouse. **a** Nuclear DNA was extracted at 16 w.p.i. from heart (H), spleen (S), lung (Lu), kidney (K) and liver (Li) of BPS HDI mice with or without persisting serum HBV markers as indicated and subjected to detection of HBV sequences by PCR. Primer sets amplifying HBsAg and HBcAg ORFs were used and GAPDH was amplified as control. HBV transgenic (Tg) mouse liver was used as positive control. **b** Nuclear DNA was extracted from liver of indicated HDI mice with indicated serum HBV marker status at indicated time points and analysed in Southern blot using HBV-specific probes. BPS plasmid DNA was used as control. **c** Digestion with one or two single-cut restriction enzyme(s) for BPS plasmid was applied as indicated before Southern blot analysis as performed in **b**. Double digestion of pUC18-based BPS HDI plasmid with *Eco*RI and *Hin*dIII releases the 1.3-fold overlength BPS genome of ~ 4.3 kb (see also Supplementary Fig. 1a). Representative results from two independently repeated experiments are shown. M, markers. w.p.i., weeks post injection.

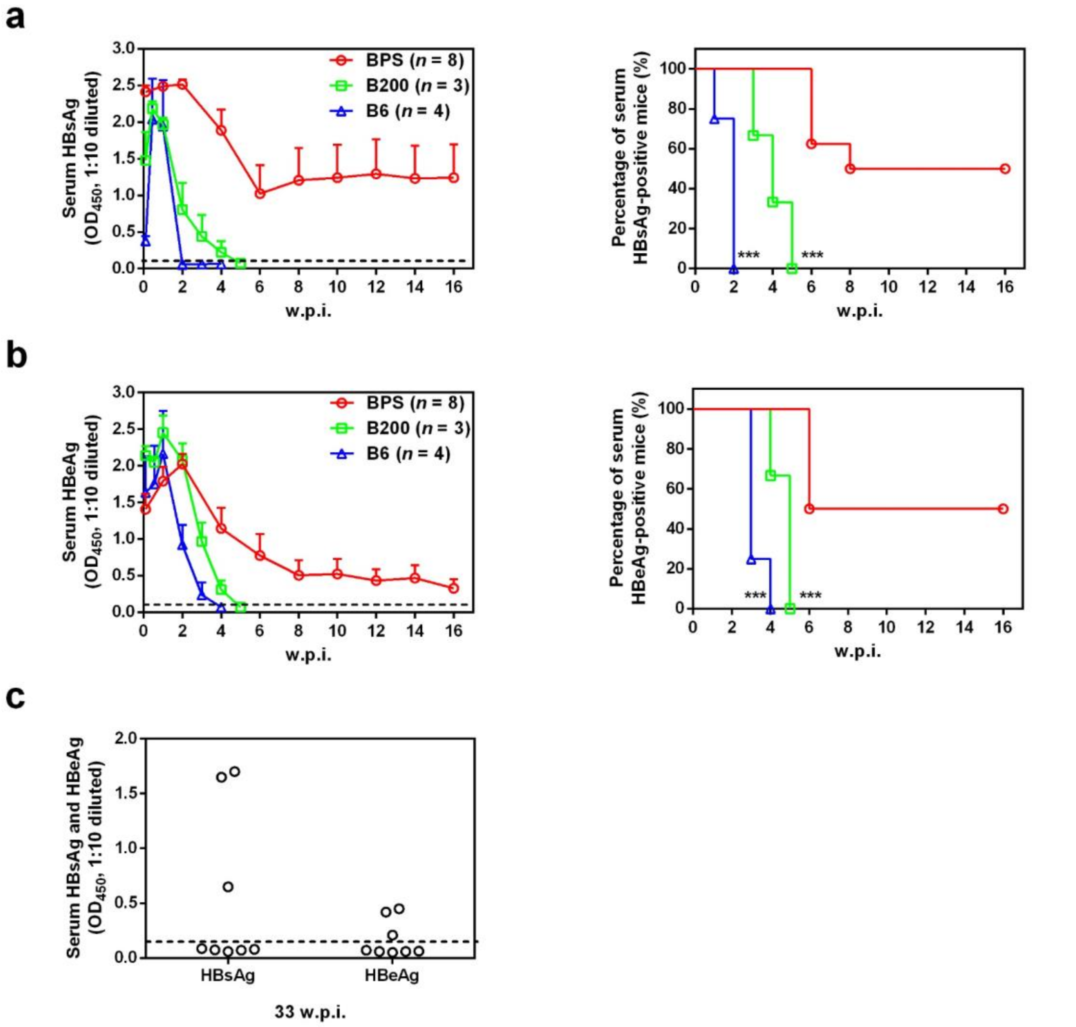

Supplementary Figure 7 Persistence of BPS in C57BL/6 mice. A single dose of 10 μg HBV replicon plasmid containing 1.3 fold overlength genome of BPS, B200 or B6 were hydrodynamically injected (HDI) into tail vein of 6-8 weeks old male C57BL/6 mice. Sera were collected at indicated time points and analysed for HBsAg **(a)** and HBeAg **(b)** using ELISA. Group means and s.e.m. within group (left) and group positivity percentage data (right) are presented with group sizes (*n*) indicated. **(c)** Serum HBV antigen levels at 33 w.p.i. were individually analysed using ELISA. Dotted lines represent cut-off thresholds. Group positivity percentages were analysed by comparing to BPS group using log-rank (Mantel-Cox) test. ***p<0.001. w.p.i., weeks post injection.

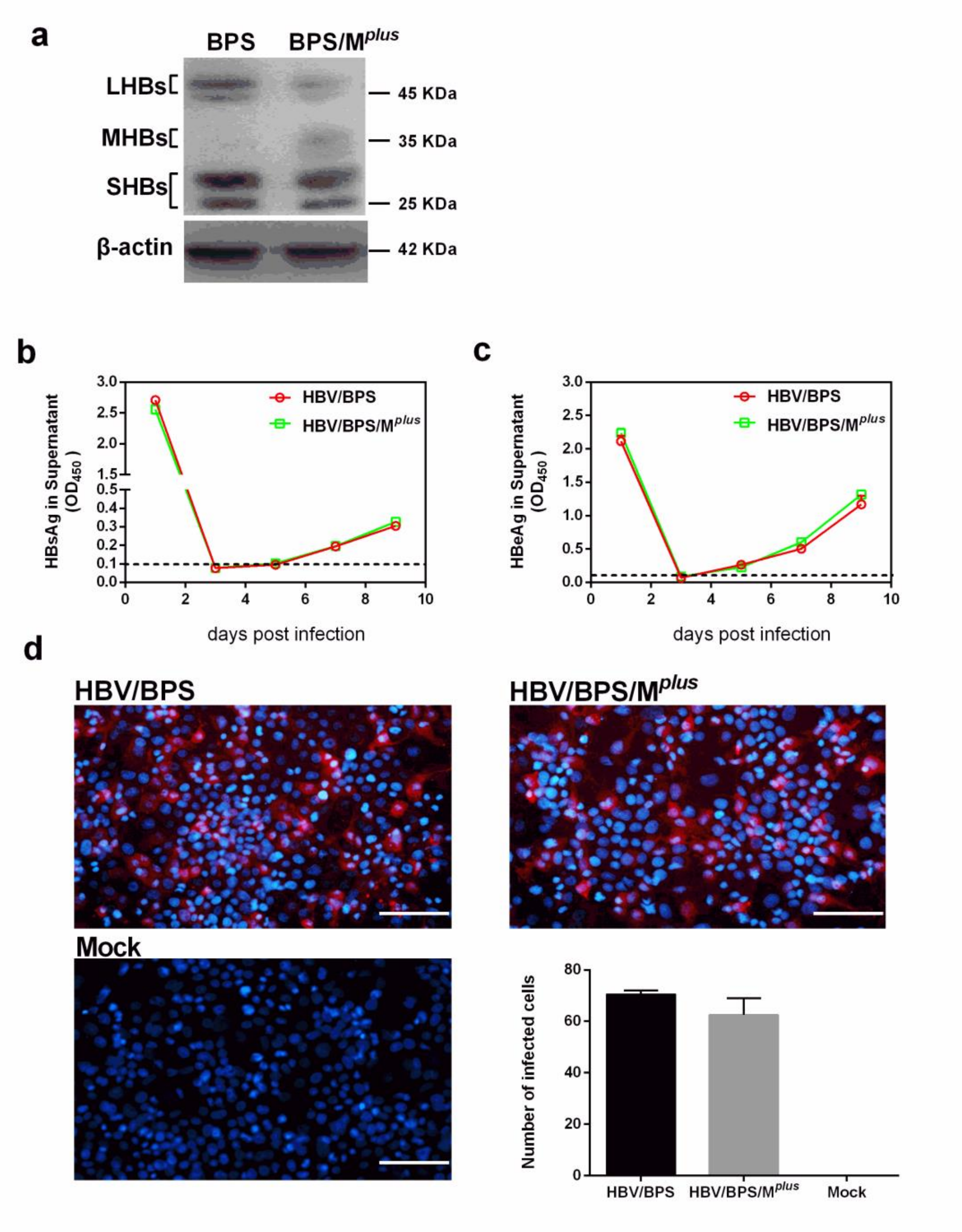

Supplementary Figure 8 HBV virions derived from BPS and BPS/M*plus* are infective. Huh-7 cells were transfected with BPS or BPS/M*plus* and intracellular HBV envelope proteins were analysed in Western blot using anti-HBsAg antibody **(a)**. Virions in transfection supernatants were concentrated and 1E7 genome equivalents of viruses were used for infecting HepG2/NTCP cells in 24-well plates. Culture medium was changed every day post infection from day 1 to day 3 and then changed every two days. The supernatants were analysed for HBsAg **(b)** and HBeAg **(c)** with ELISA. **d** Intracellular HBcAg was detected using immunofluorescence. Number of infected cells were counted using 10 randomly selected view fields at 100 fold magnification for each treatment and means and s.e.m. of the 10 views are presented (bottom right). The infection experiments were independently repeated twice and representative results are presented. Dotted lines represent cut-off thresholds. Scar bars, 100μm.

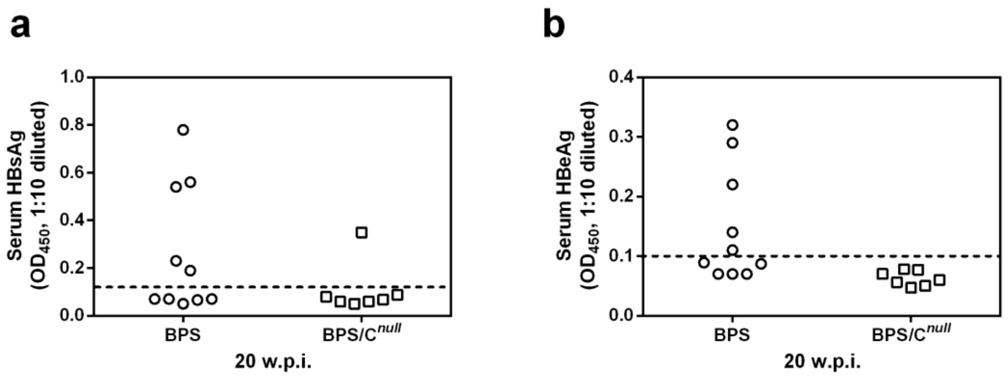

Supplementary Figure 9 BPS/C*null* was cleared more quickly than BPS. Sera from BALB/c mice injected with BPS/C*null* and BPS as shown in Fig. 3d and 3e were individually analysed for HBsAg **(a)** and HBeAg **(b)** at 20 w.p.i. using ELISA. Dotted lines represent cut-off thresholds. w.p.i., weeks post injection.

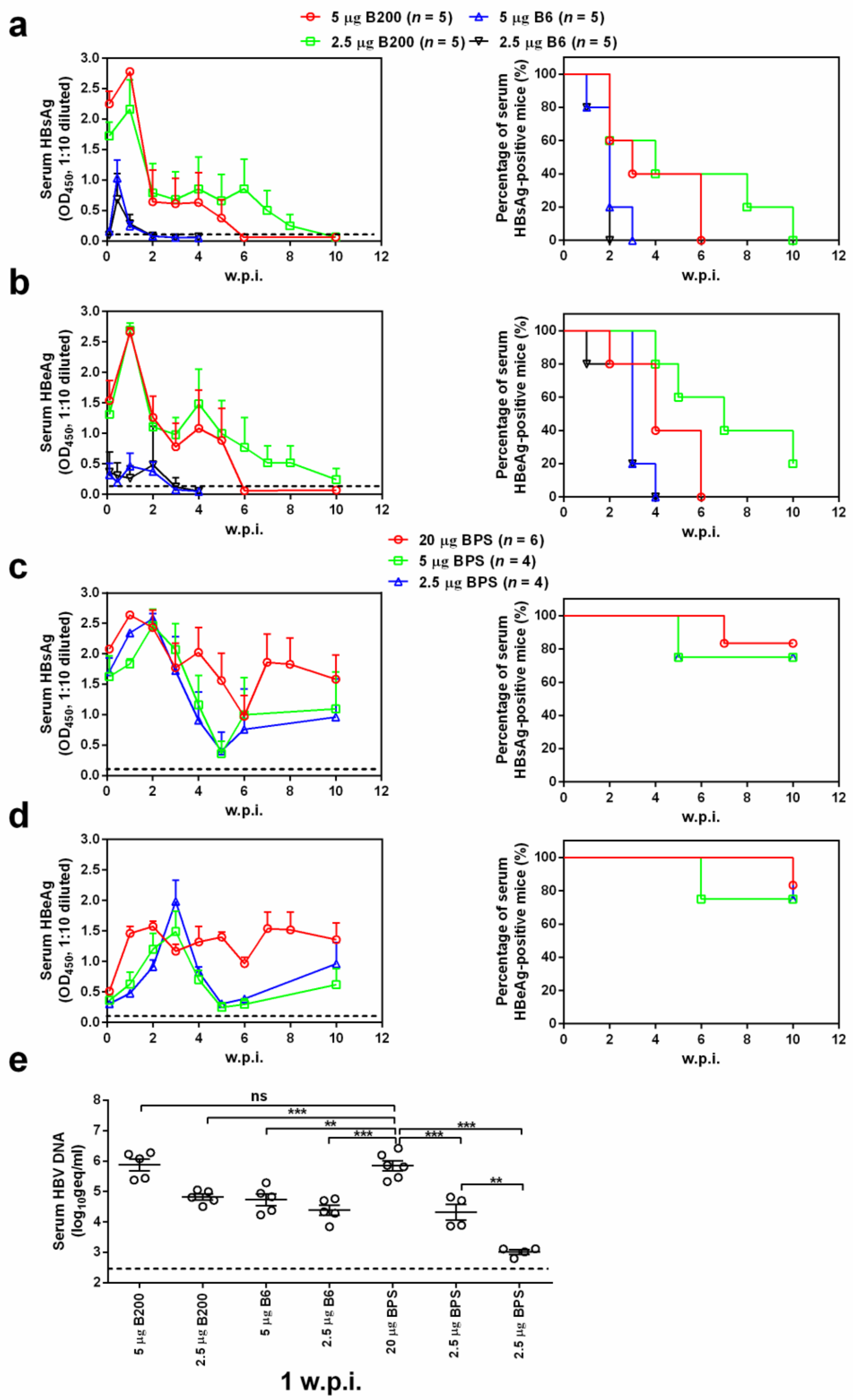

Supplementary Figure 10 Effects of injection dosage on outcome of B6, B200 and BPS HDI. A single dose of indicated amount of B6 or B200 **(a&b)** or BPS **(c&d)** replicon plasmid was hydrodynamically injected (HDI) into tail vein of 6-8 weeks old male BALB/c mice. Sera were collected at indicated time points and analysed for HBsAg **(a&c)** and HBeAg **(b&e)** using ELISA. Group means and s.e.m. within group are presented with group sizes (*n*) indicated and dotted lines represent cut-off thresholds. Group positivity percentages are shown in the right panels. Serum

HBV DNA levels at 1 w.p.i. were analysed using quantitative realtime PCR **(e)**. Individual titers, group means and s.e.m. within group are plotted, and the latter were compared using unpaired two-tailed *t*-test. ns, not significant; **p<0.01; ***p<0.001. geq, genome equivalents. w.p.i., weeks post injection.

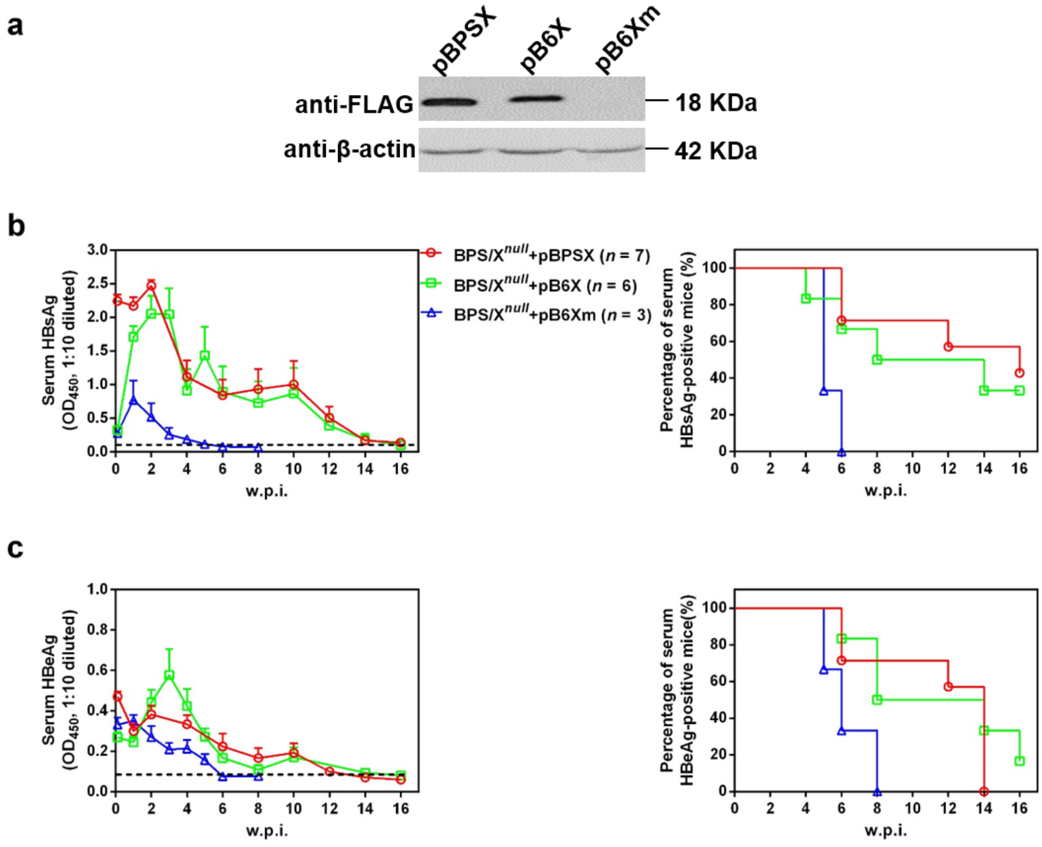

Supplementary Figure 11 Effects of HBx on BPS persistence. BPS mutant with obliterated X ORF (BPS/X^{null}) was *trans*-complemented with BPS- or B6-derived X expression plasmids, pBPSX or pB6X, and co-injected into BALB/c mice. pB6X-derived mutant pB6Xm with obliterated X ORF was used as control. Expression of FLAG-tagged X from pBPSX and pB6X was confirmed by Western blot **(a)** following transfection of Huh-7 cells. Sera from HDI mice were collected at indicated time points and analysed for HBsAg **(b)** and HBeAg **(c)** using ELISA. Group means and s.e.m. within group are presented with group sizes (*n*) indicated. Dotted lines represent cut-off thresholds. w.p.i., weeks post injection.

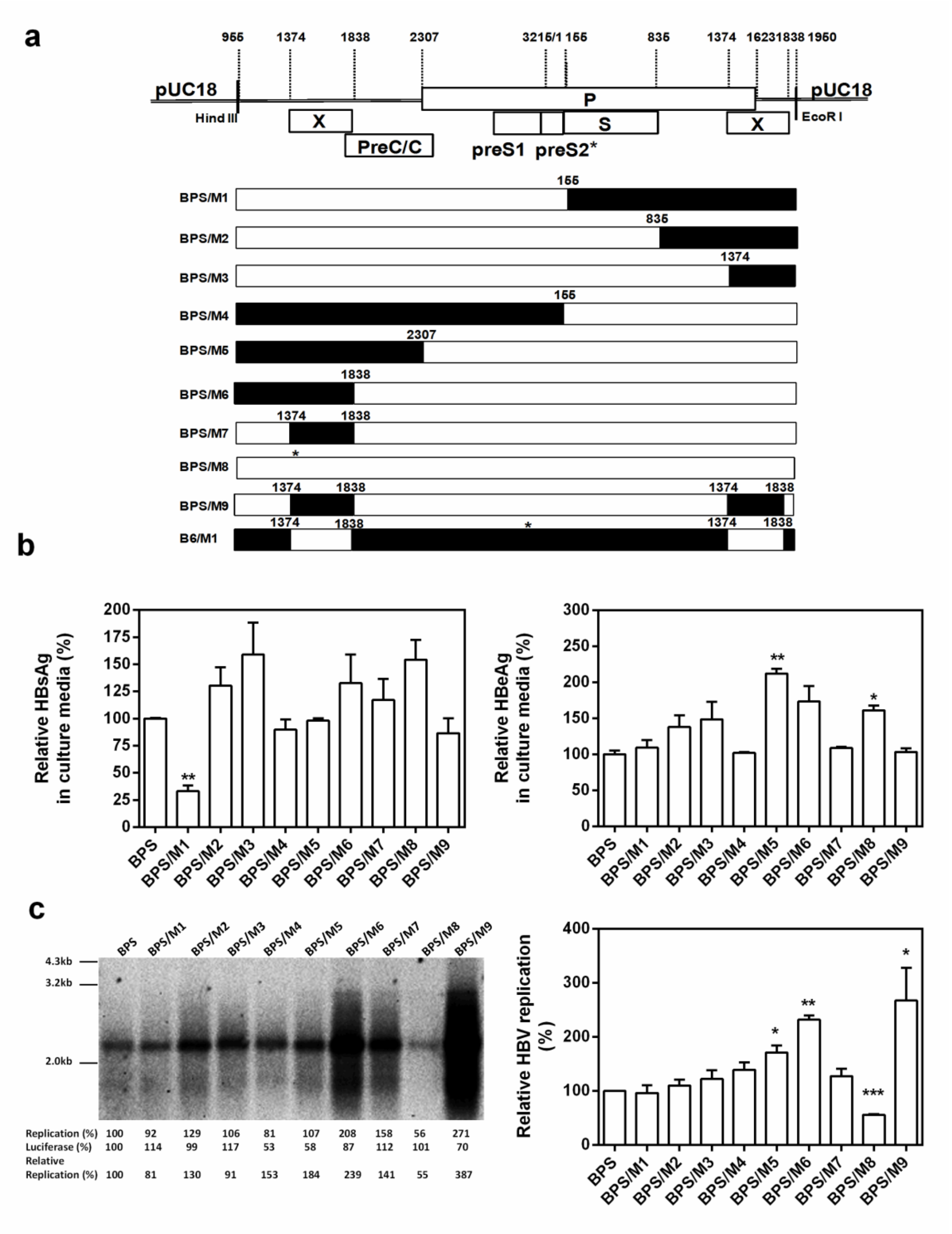

Supplementary Figure 12 Chimeric constructs between BPS and B6 and additional BPS mutants. **a** Schematic representation of the chimeras and mutants. BPS- and B6-derived segments are represented by open and solid boxes respectively. Junctions were marked with numbers indicating their positions on HBV genome (top). *, X^{null} (Q8ter) mutation in the upstream X ORF of BPS/M8 and M1T mutation in preS2 ORF of B6/M1. Culture supernatants of transfected Huh-7 cells were analysed for HBsAg and HBeAg **(b)** by ELISA. Intracellular capsid-associated HBV DNA was analysed in Southern blot and HBV replication intermediates were quantified using densitometry scanning **(c)**. Transfection efficiency was normalized using co-transfected pGL3 plasmid and means and s.e.m. calculated using normalized data from 2 independent biological replicates taking

BPS measurements as 100% are presented in **a**, **b** and **c** (right). Representative Southern blot results are shown in **c** (left). Statistical significances are calculated by comparing to BPS data using unpaired two-tailed *t*-test. *p<0.05; **p<0.01; ***p<0.001.

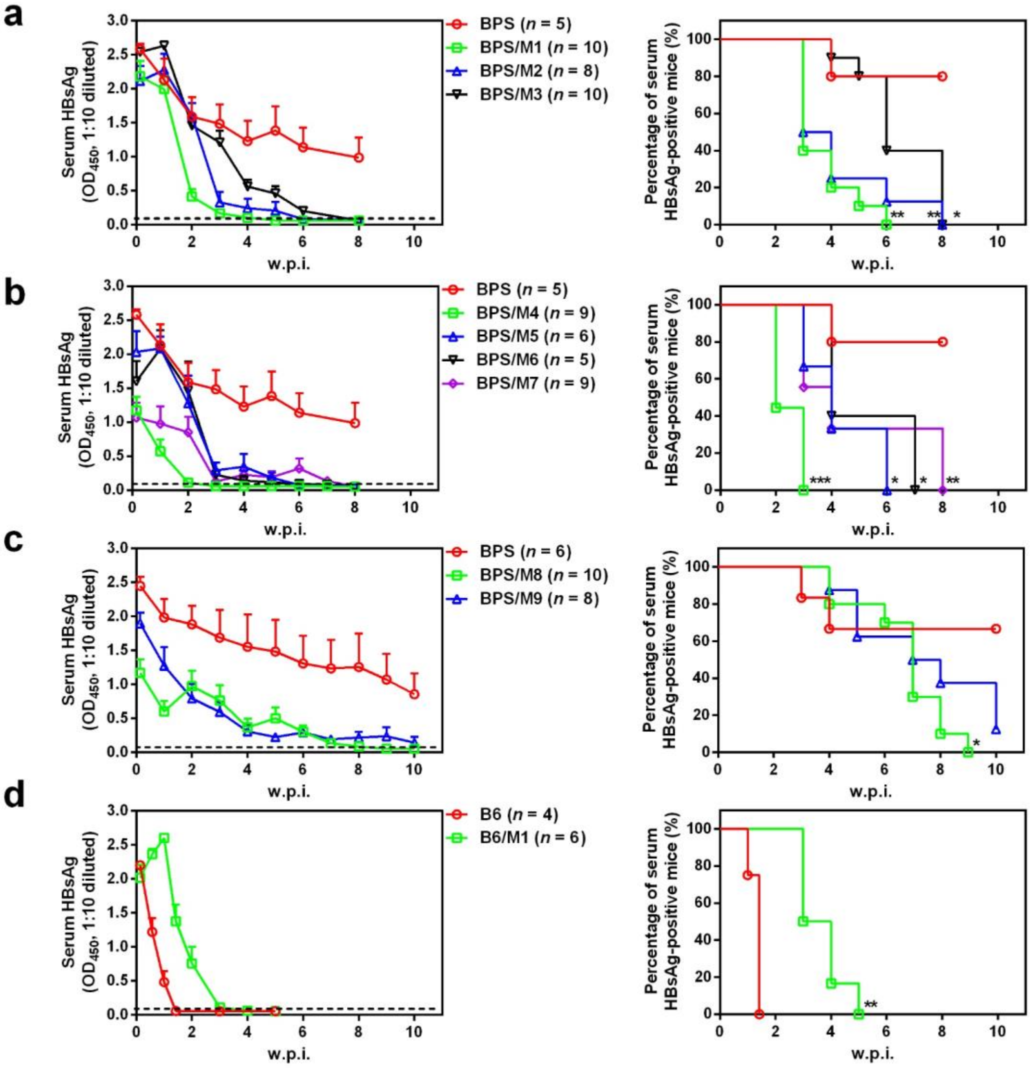

Supplementary Figure 13 Outcome of HDI using chimeric constructs between BPS and B6 and additional BPS mutants. Sera collected from BALB/c mice injected with indicated constructs were analysed for HBsAg. Group means and s.e.m. within group (left) and group positivity data (right) are presented with group sizes (*n*) indicated. Dotted lines represent cut-off threshold. Group positivity data are compared against BPS and statistical significance calculated using log-rank (Mantel-Cox). **p*<0.05; ***p*<0.01; ****p*<0.001. w.p.i., weeks post injection.

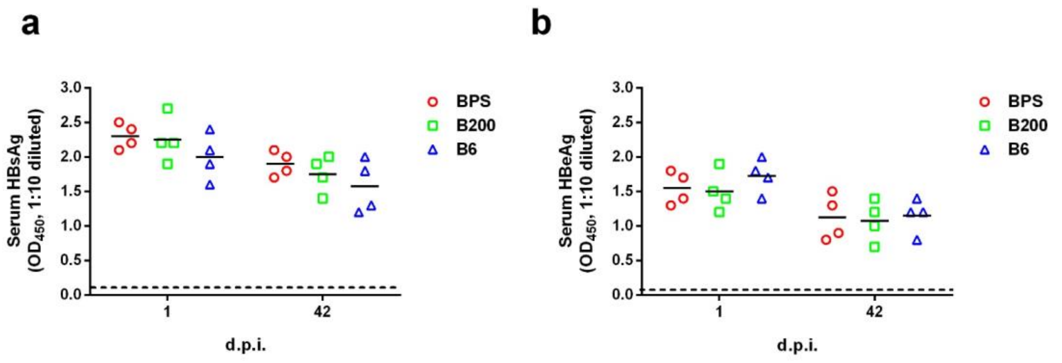

Supplementary Figure 14 Host immunodeficiency results in HBV persistence in HDI mice. Athymic nude mice were injected with replicon plasmids derived from indicated genotype B HBV strains. Sera were collected at indicated time points and individually analysed for HBsAg **(a)** and HBeAg **(b)** in ELISA. Data for individual mice and group means are presented. Dotted lines represent cut-off thresholds. d.p.i., days post injection.

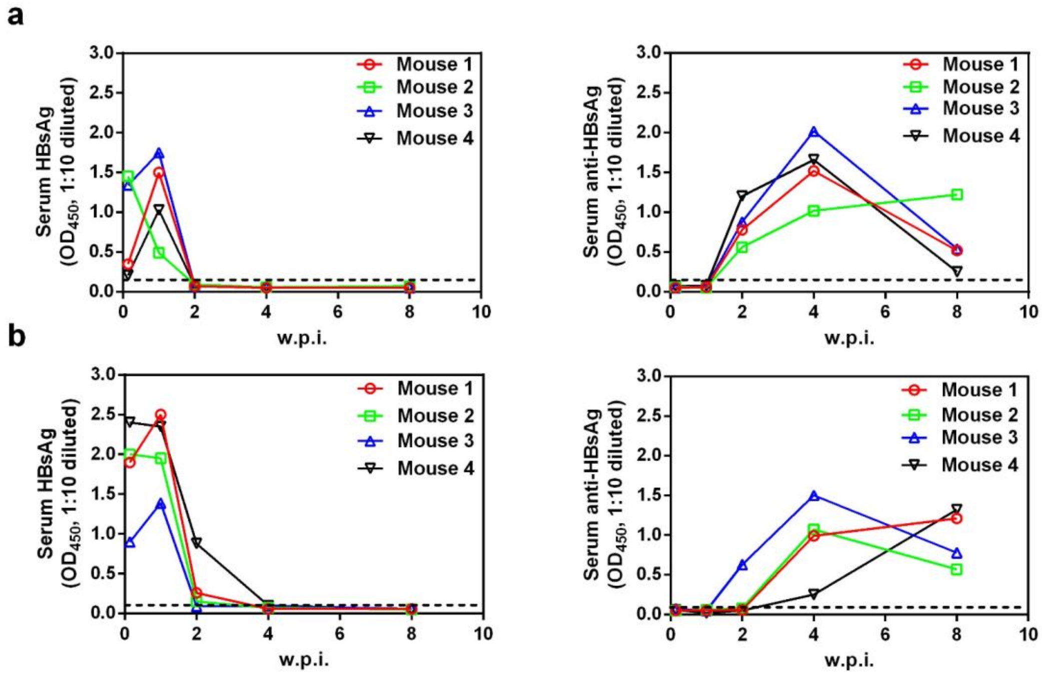

Supplementary Figure 15 Clearance of HBV by HDI mice correlates with HBsAg to HBsAb seroconversion. Sera from B6 **(a)** and B200 **(b)** HDI BALB/c mice were collected at indicated time points and individually analysed for HBsAg (left) and HBsAb (right) in ELISA. Dotted lines represent cut-off thresholds. w.p.i., weeks post injection.

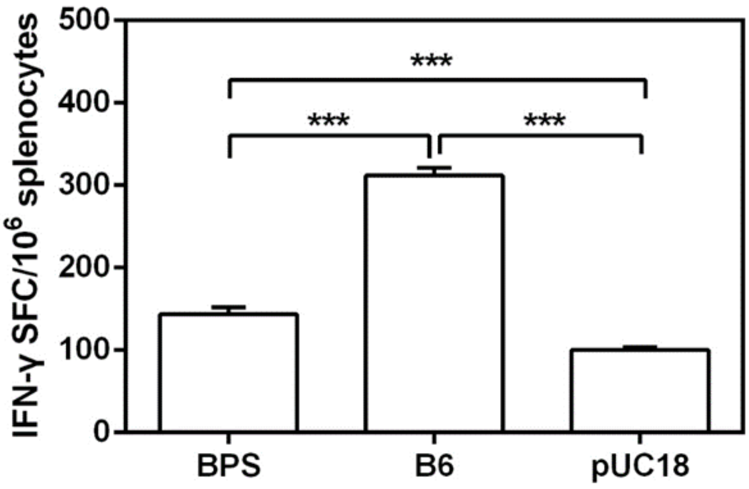

Supplementary Figure 16 ELISPOT analysis of HBsAg-specific spleen T cell in HDI BALB/c mice. BPS, B6 or pUC18 HDI BALB/c mice (*n* = 3) were sacrificed at 5 weeks post injection. Only BPS mice were positive for serum HBsAg by this time. Splenocytes were prepared and stimulated with 15 μg/ml recombinant HBsAg for 18 hours. Gamma interferon (IFN-γ)-secreting cells were measured using commercial IFN-γ ELISPOT assay kit (eBioscience) and spots were analysed using iSpot ELISPOT Reader (AID, Germany). Splenocytes from each mouse were analysed in duplicates. Group means and s.e.m. within group are presented. SFC: spot-forming cells. BPS and B6 mice data are compared against pUC18 mice and statistical significance calculated using unpaired two-tailed *t*-test. ***p<0.001.

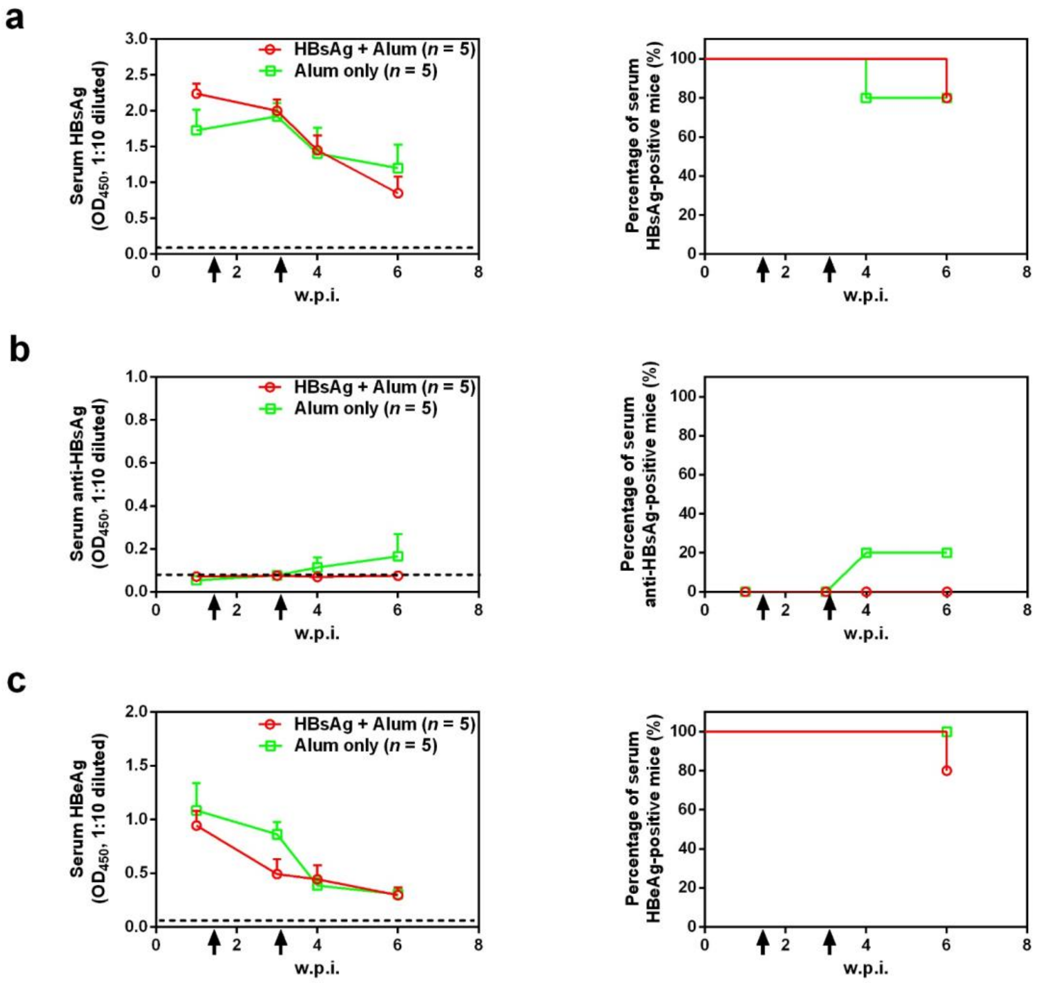

Supplementary Figure 17 Lack of effects of post-exposure HBsAg immunization on BPS persistence in HDI mice. BPS HDI BALB/c mice were intramuscularly injected with recombinant HBsAg vaccine emulsified in alum, or alum only, at 10 and 20 days post BPS injection (arrows). Sera were collected at indicated time points and analysed for HBsAg **(a)**, HBsAb **(b)** and HBeAg **(c)** using ELISA. Group means (*n* = 5) and s.e.m. within group are presented. Dotted lines represent cut-off thresholds. w.p.i., weeks post injection of BPS.

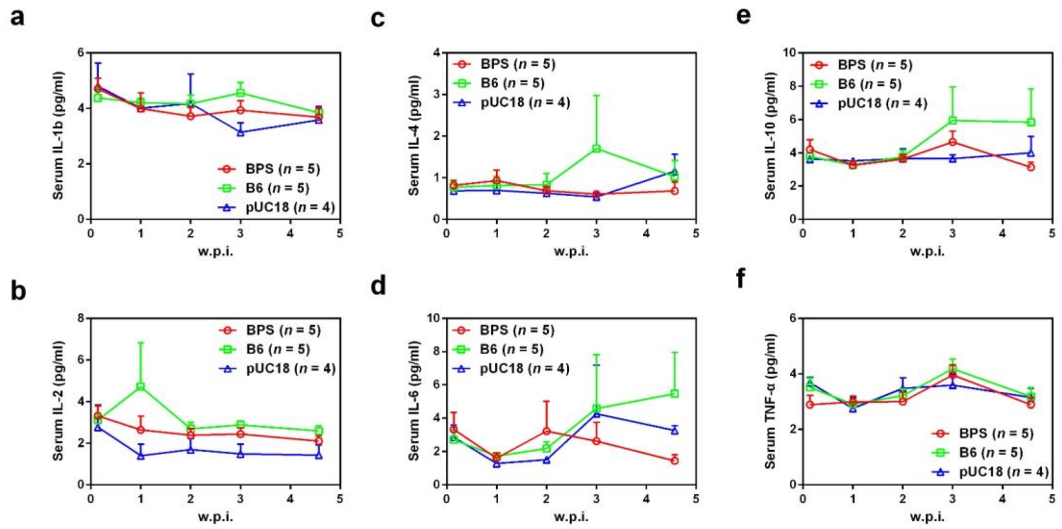

Supplementary Figure 18 Serum cytokine profiling of HBV HDI mice. Sera from BPS, B6 and pUC18 empty vector HDI BALB/c mice were collected at indicated time points and levels of selected cytokines quantitated using Multiplex-based assay. Group means and s.e.m. within group are presented with group size (*n*) indicated. w.p.i., weeks post injection.

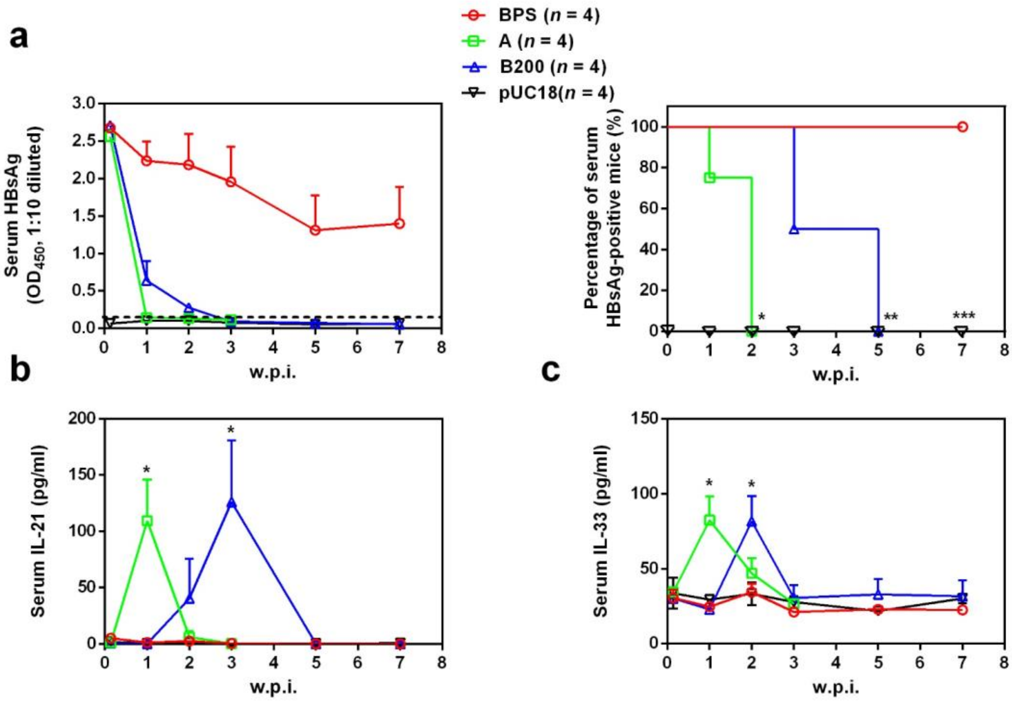

Supplementary Figure 19 Post-exposure IL-21 and IL-33 responses in HBV HDI mice. Sera from BPS, genotype A, B200 and pUC18 empty vector HDI BALB/c mice (*n* = 4) were collected at indicated time points. Serum HBsAg **(a)** was measured using ELISA and levels of IL-21 **(b)** and IL-33 **(c)** quantitated using Multiplex-based assay. Dotted lines represent cut-off thresholds. Group means and s.e.m. within group are presented. Group positivity data for serum HBsAg are also presented (**a**, right) and compared against BPS and statistical significance calculated using log-rank (Mantel-Cox). Interleukin data of HBV HDI mice are compared against pUC18 mice and statistical significance calculated using unpaired two-tailed *t*-test. *p<0.05; **p<0.01; ***p<0.001. w.p.i., weeks post injection.

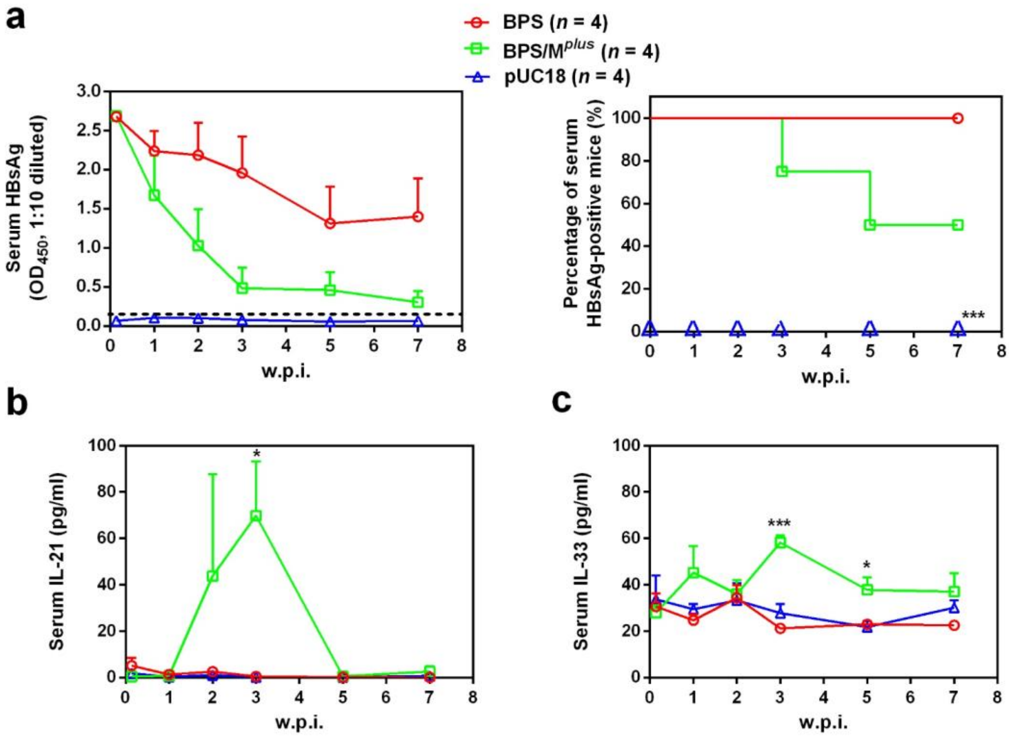

Supplementary Figure 20 Post-exposure IL-21 and IL-33 responses in BPS/M^{plus} HDI mice. Sera from BPS, BPS/M^{plus} and pUC18 empty vector HDI BALB/c mice (n = 4) were collected at indicated time points. Serum HBsAg **(a)** was measured using ELISA and levels of IL-21 **(b)** and IL-33 **(c)** quantitated using Multiplex-based assay. Dotted lines represent cut-off thresholds. Group means and s.e.m. within group are presented. Group positivity data for serum HBsAg are also presented (**a**, right) and compared against pUC18 mice and statistical significance calculated using log-rank (Mantel-Cox). Interleukin data are compared against pUC18 mice and statistical significance calculated using unpaired two-tailed *t*-test. *$p<0.05$; ***$p<0.001$. w.p.i., weeks post injection.

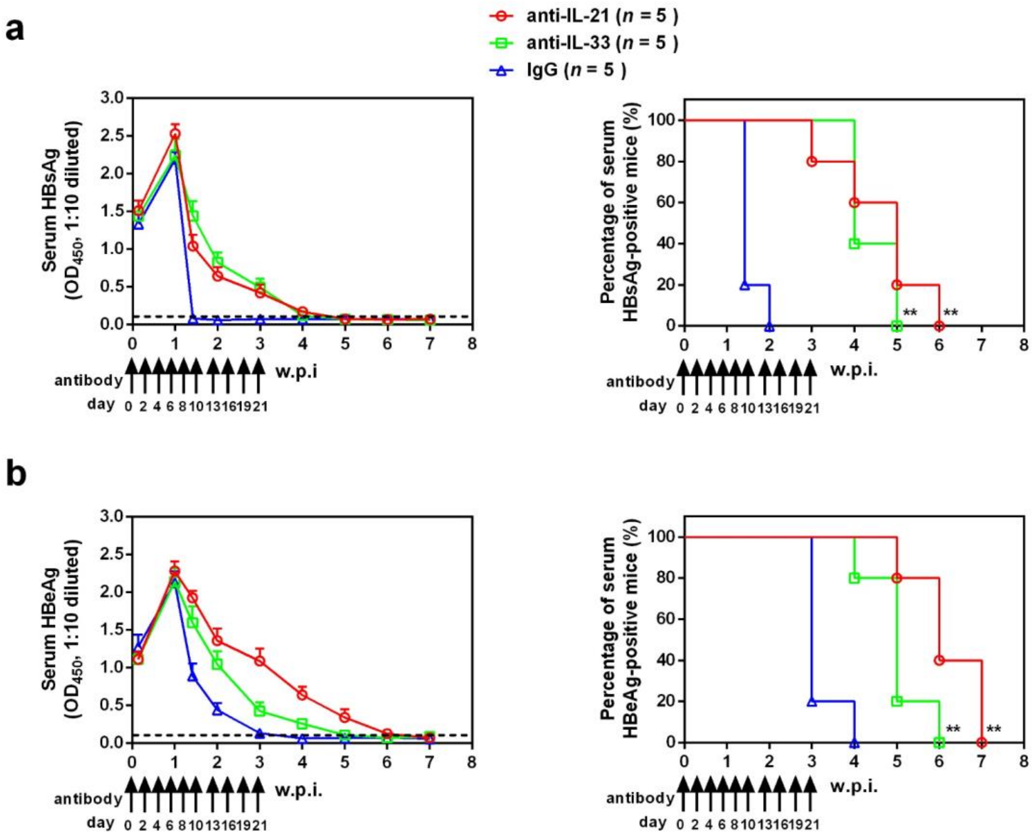

Supplementary Figure 21 Treatment with antibodies against IL-21 or IL-33 delayed HBV clearance in B6 HDI mice. Ten micrograms of rat monoclonal antibodies against mouse IL-21 or IL-33, or normal IgG control were injected intraperitoneally into BALB/c mice (*n* = 5) at indicated time points (arrows) after B6 HDI procedure. Sera were collected at indicated time points and analysed for HBsAg **(a)** and HBeAg **(b)** using ELISA. Group means and s.e.m. within group are presented (left). Group positivity data (right) are compared against IgG injected mice and statistical significance calculated using log-rank (Mantel-Cox). $^{**}p<0.01$. Dotted lines represent cut-off thresholds. w.p.i., weeks post injection.

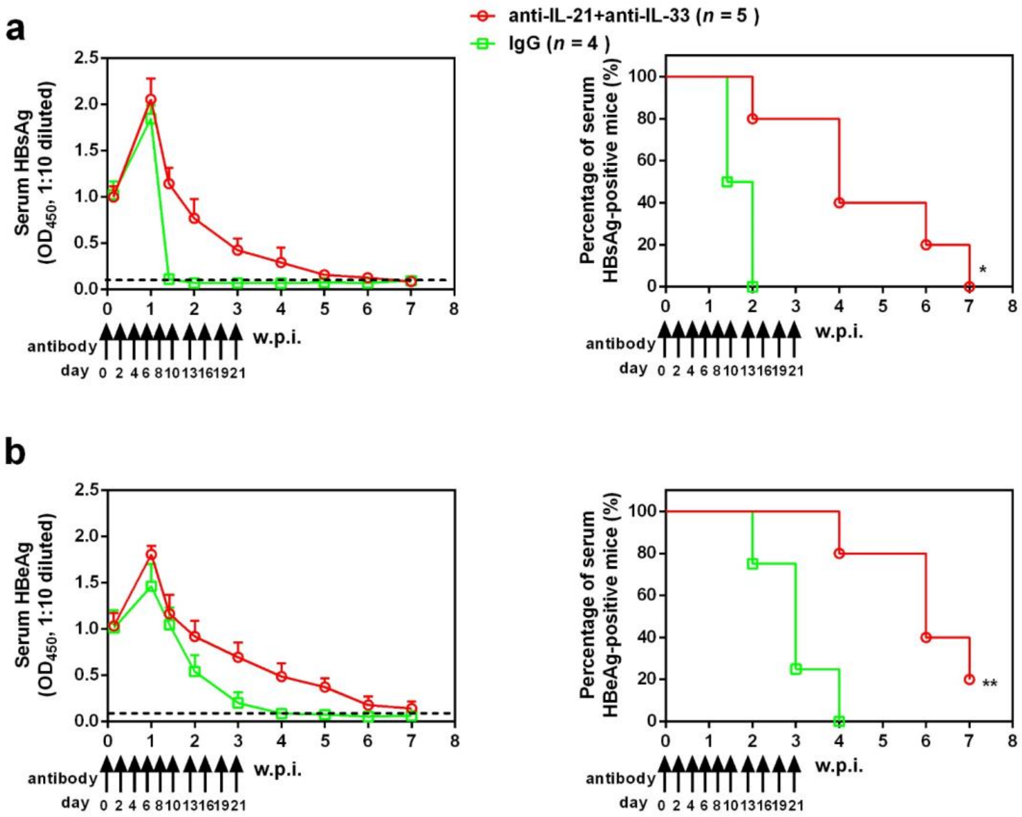

Supplementary Figure 22 Treatment with combined IL-21 and IL-33 antibodies delayed HBV clearance in B6 HDI mice. Ten micrograms each of rat monoclonal antibodies against mouse IL-21 and IL-33, or 20 μg normal IgG control were injected intraperitoneally into BALB/c mice at indicated time points (arrows) after B6 HDI procedure. Sera were collected at indicated time points and analysed for HBsAg **(a)** and HBeAg **(b)** using ELISA. Group means and s.e.m. within group are presented (left) with group sizes (*n*) indicated. Group positivity data (right) are compared against IgG injected mice and statistical significance calculated using log-rank (Mantel-Cox). *p<0.05; **p<0.01. Dotted lines represent cut-off thresholds. w.p.i., weeks post injection.

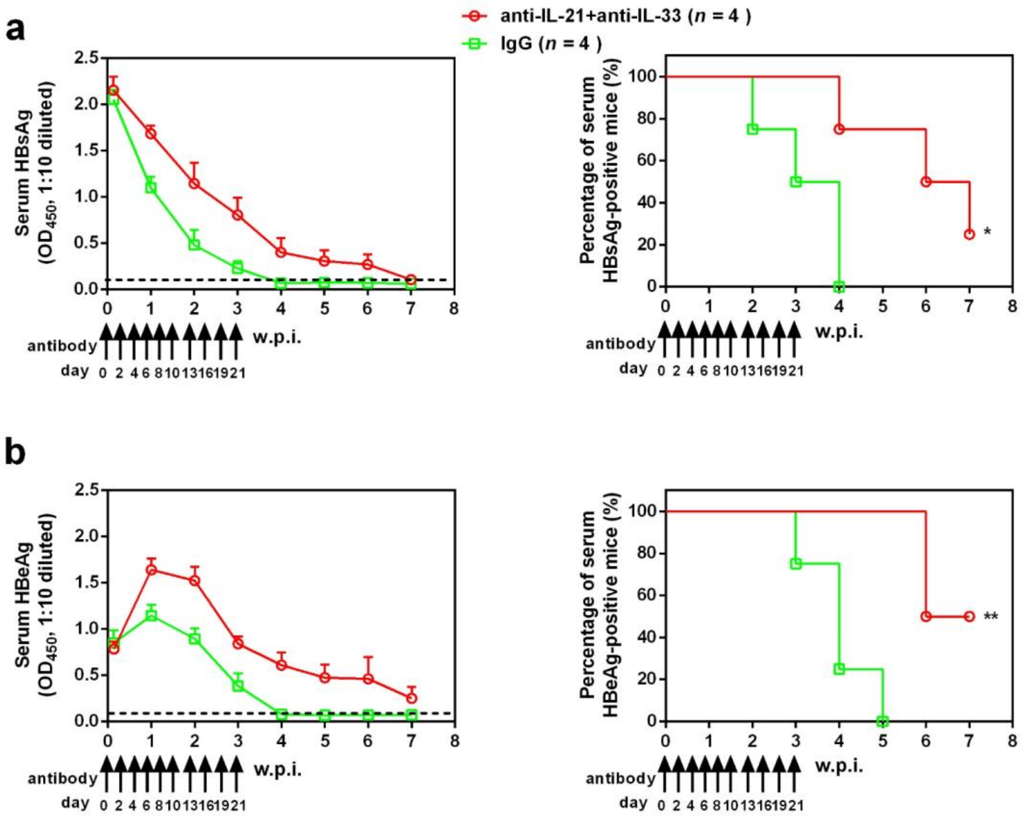

Supplementary Figure 23 Treatment with combined IL-21 and IL-33 antibodies delayed HBV clearance in B200 HDI mice. Ten micrograms each of rat monoclonal antibodies against mouse IL-21 and IL-33, or 20 μg normal IgG control were injected intraperitoneally into BALB/c mice at indicated time points (arrows) after B200 HDI procedure. Sera were collected at indicated time points and analysed for HBsAg **(a)** and HBeAg **(b)** using ELISA. Group means and s.e.m. within group are presented (left) with group sizes (*n*) indicated. Group positivity data (right) are compared against IgG and statistical significance calculated using log-rank (Mantel-Cox). *$p<0.05$; **$p<0.01$. Dotted lines represent cut-off thresholds. w.p.i., weeks post injection.

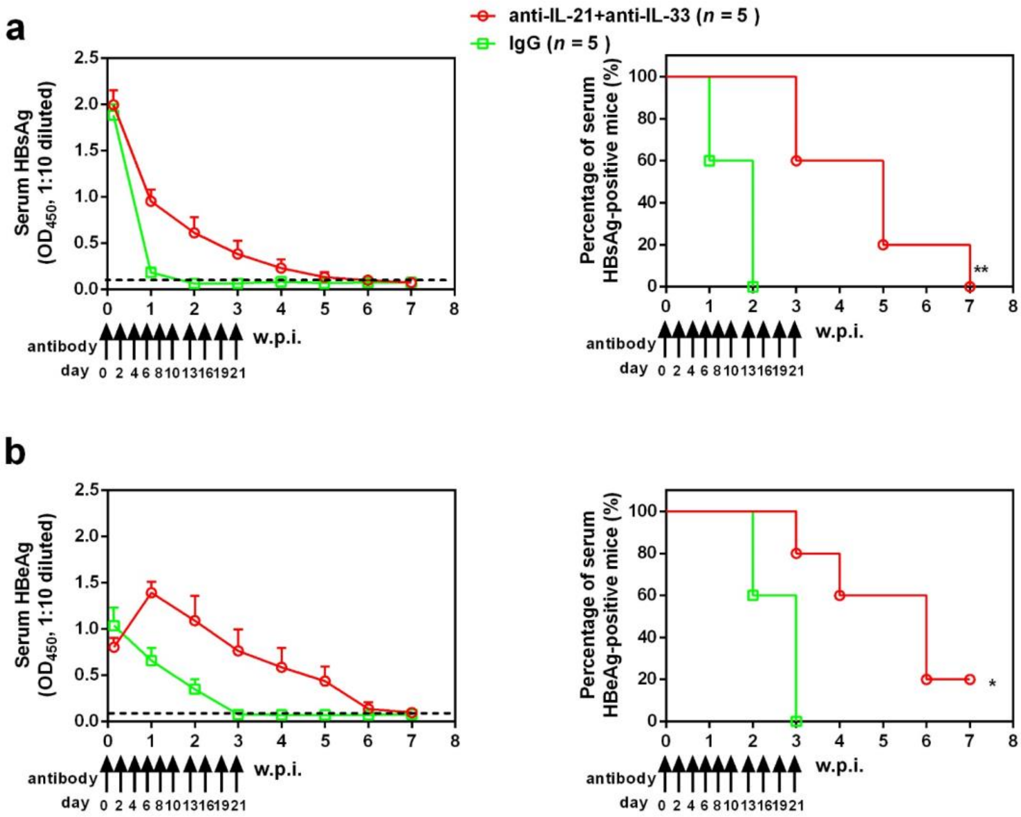

Supplementary Figure 24 Treatment with combined IL-21 and IL-33 antibodies delayed HBV clearance in genotype A HDI mice. Ten micrograms each of rat monoclonal antibodies against mouse IL-21 and IL-33, or 20 μg normal IgG control were injected intraperitoneally into BALB/c mice at indicated time points (arrows) after genotype A HBV HDI procedure. Sera were collected at indicated time points and analysed for HBsAg **(a)** and HBeAg **(b)** using ELISA. Group means and s.e.m. within group are presented (left) with group sizes (*n*) indicated. Group positivity data (right) are compared against IgG and statistical significance calculated using log-rank (Mantel-Cox). *$p<0.05$; **$p<0.01$. Dotted lines represent cut-off thresholds. w.p.i., weeks post injection.

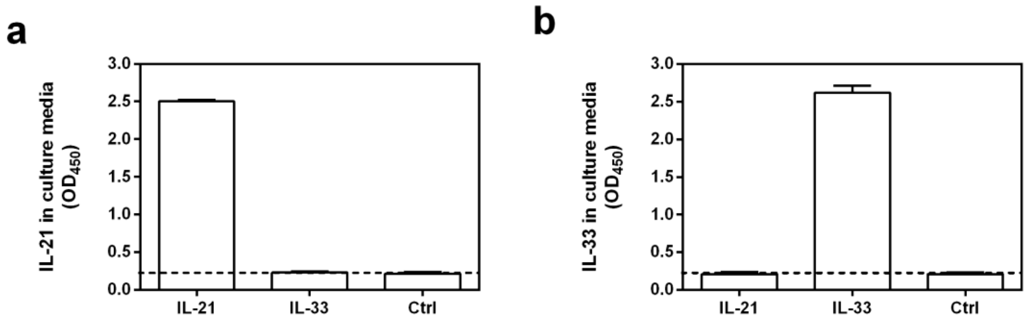

Supplementary Figure 25 Validation of IL-21 and IL-33 expression plasmids in transfected Huh-7 cells. Plasmids expressing murine IL-21 or IL-33 were transfected into cultured Huh-7 cells in triplicates and 48 hours post transfection, IL-21 **(a)** and IL-33 **(b)** in culture supernatants were detected using ELISA. Empty vector plasmid was used as control. Means and s.e.m..of triplicates are presented. Dotted lines represent cut-off thresholds.

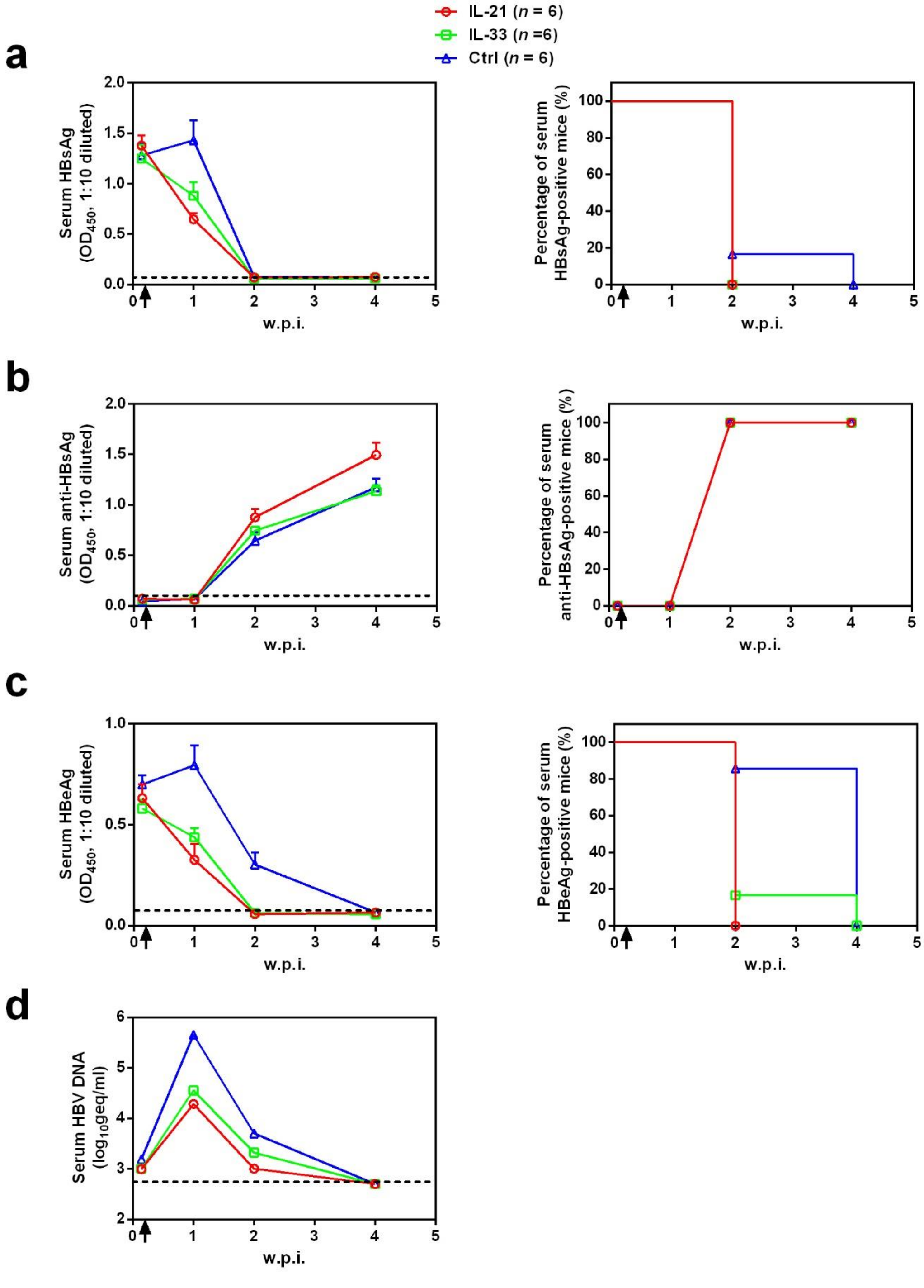

Supplementary Figure 26 Injection with IL-21 and IL-33 expression plasmid facilitated HBV clearance in B6 HDI mice. Plasmids expressing murine IL-21 or IL-33, or control vector were hydrodynamically injected into BALB/c mice (*n*= 6) 24 hours after B6 HDI procedure (arrow). Sera were collected at indicated time points and analysed for HBsAg **(a)**, HBsAb **(b)** and HBeAg **(c)** using ELISA. Group means and s.e.m. within group are presented. Pooled sera from each group were used for detecting HBV DNA using commercial quantitative assay **(d)**. Dotted lines represent cut-off thresholds (**a-c**) or detection limit (**d**). w.p.i., weeks post injection. geq, genome equivalent.

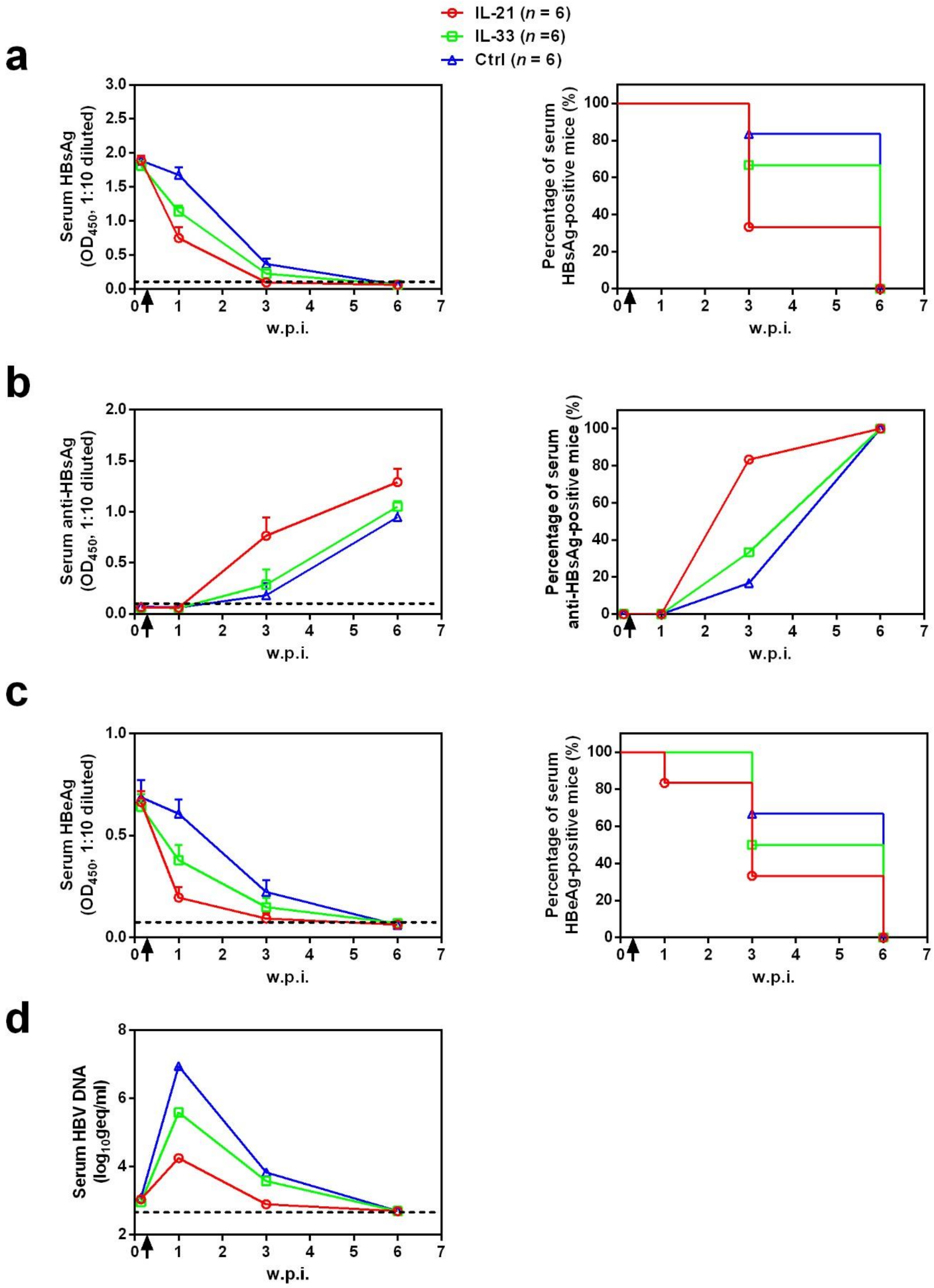

Supplementary Figure 27 Injection with IL-21 and IL-33 expression plasmid facilitated HBV clearance in B200 HDI mice. Plasmids expressing murine IL-21 or IL-33 were hydrodynamically injected into BALB/c mice (*n* = 6) 24 hours after B200 HDI procedure (arrow). Sera were collected at indicated time points and analysed for HBsAg **(a)**, HBsAb **(b)** and HBeAg **(c)** using ELISA. Group means and s.e.m. within group are presented. Pooled sera from each group were used for detecting HBV DNA using commercial quantitative assay **(d)**. Dotted lines represent cut-off thresholds (**a-c**) or detection limit (**d**). w.p.i., weeks post injection. geq, genome equivalent.

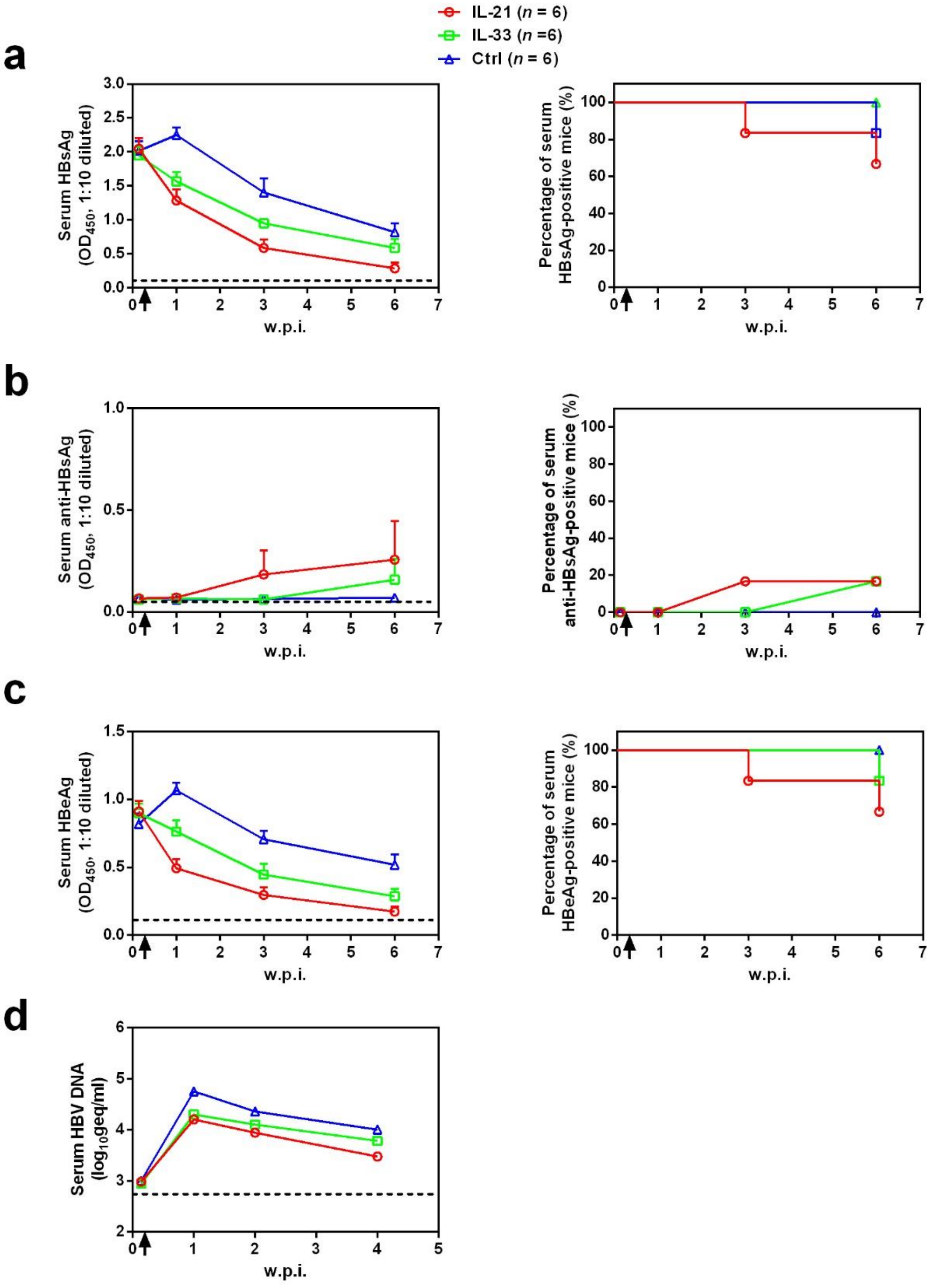

Supplementary Figure 28 Injection of BPS HDI mice in acute phase with IL-21 and IL-33 expression plasmid decreased HBV serum antigens without causing clearance. Plasmids expressing murine IL-21 or IL-33 were hydrodynamically injected into BALB/c mice (*n* = 6) 24 hours after BPS HDI procedure (arrow). Sera were collected at indicated time points and analysed for HBsAg **(a)**, HBsAb **(b)** and HBeAg **(c)** using ELISA. Group means and s.e.m. within group are presented. Pooled sera from each group were used for detecting HBV DNA using commercial quantitative assay **(d)**. Dotted lines represent cut-off thresholds (**a-c**) or detection limit (**d**). w.p.i., weeks post injection. geq, genome equivalent.

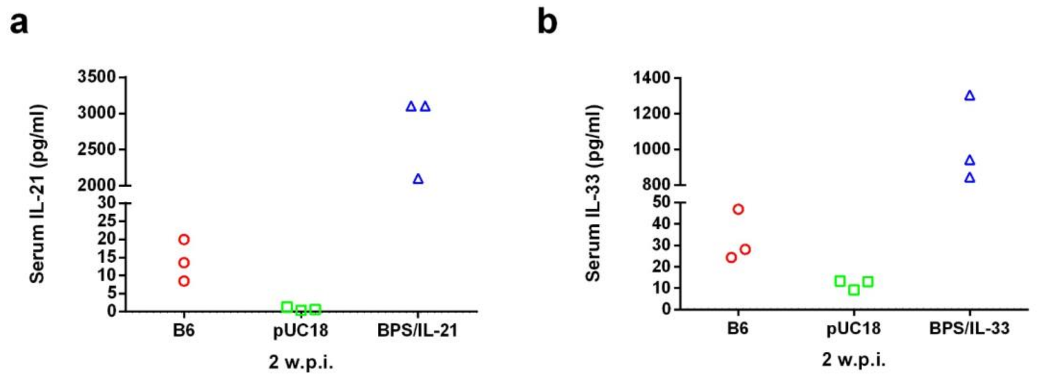

Supplementary Figure 29 Quantitative analysis of exogenous and endogenous IL-21 and IL-33 levels in HDI mice. Sera from BPS (*n* = 3), B6 (*n* = 3) and pUC18 empty vector (*n* = 3) HDI BALB/c mice were collected at 2 weeks post injection (w.p.i.) and levels of IL-21 **(a)** and IL-33 **(b)** were quantitated using Multiplex-based assay. Serum HBsAg positive BPS HDI mice at 4 w.p.i. were injected with IL-21 (**a**) or IL-33 (**b**) expression plasmid and serum levels of IL-21 or IL-33 were similarly measured 2 weeks later.

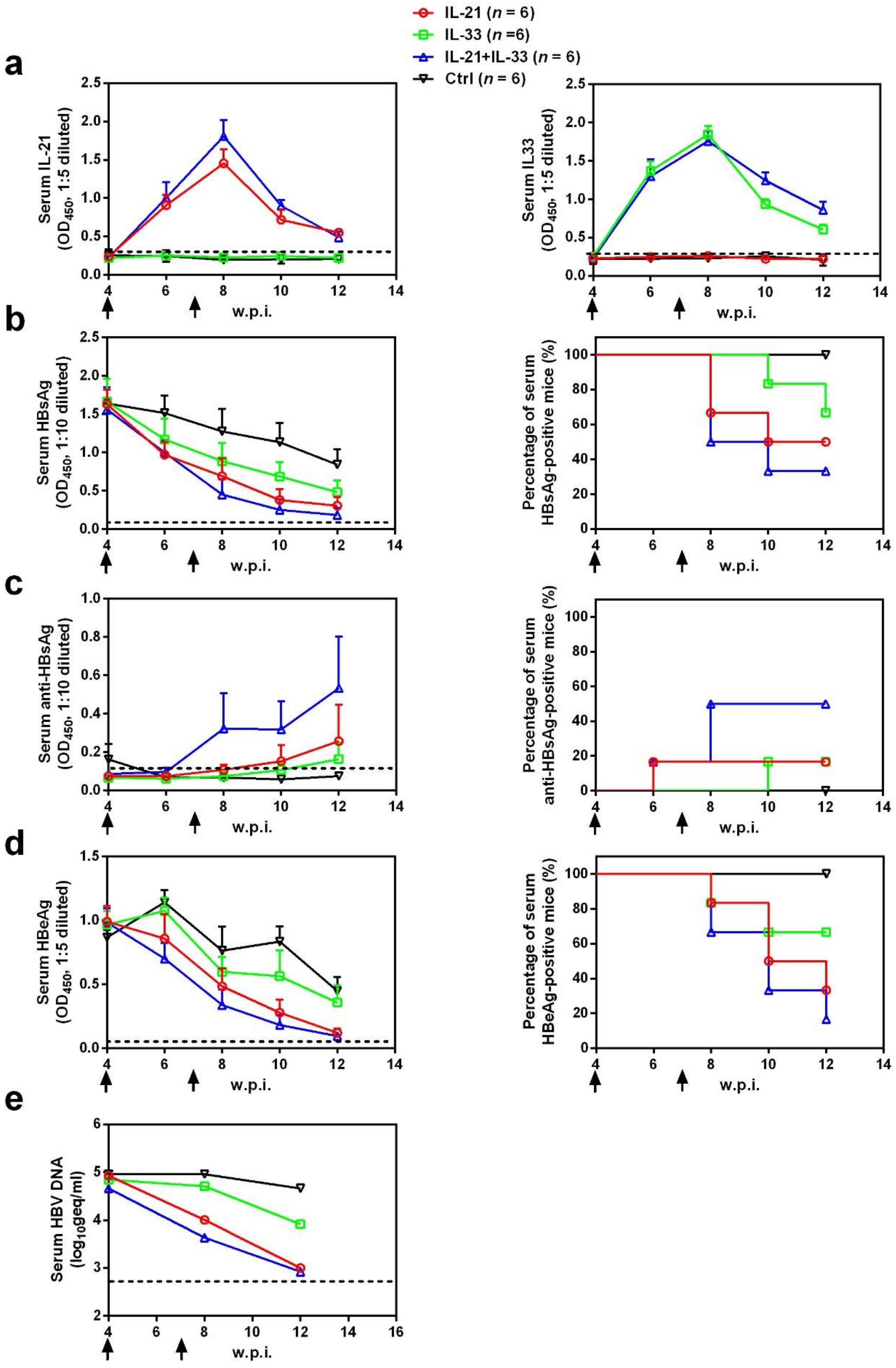

Supplementary Figure 30 Comparison of combined IL-21 and IL-33 plasmids injection with mono injections in clearing established BPS persistence. Serum HBsAg positive BPS HDI BALB/c mice (*n* = 6) were injected with indicated plasmids at 4 and 7 w.p.i (arrows). Sera were collected at indicated time points and analysed for IL-21 and IL-33 **(a)**, HBsAg **(b)**, HBsAb **(c)**,

and HBeAg **(d)** using ELISA. Group means and s.e.m. within group are presented and group positivity data for HBsAg, HBsAb, and HBeAg are also presented (**b-d**, right)**.** Pooled sera from each group were used for detecting HBV DNA using commercial quantitative assay **(e)**. Dotted lines represent cut-off thresholds (**a-d**) or detection limit (**e**). w.p.i., weeks post injection. geq, genome equivalent.

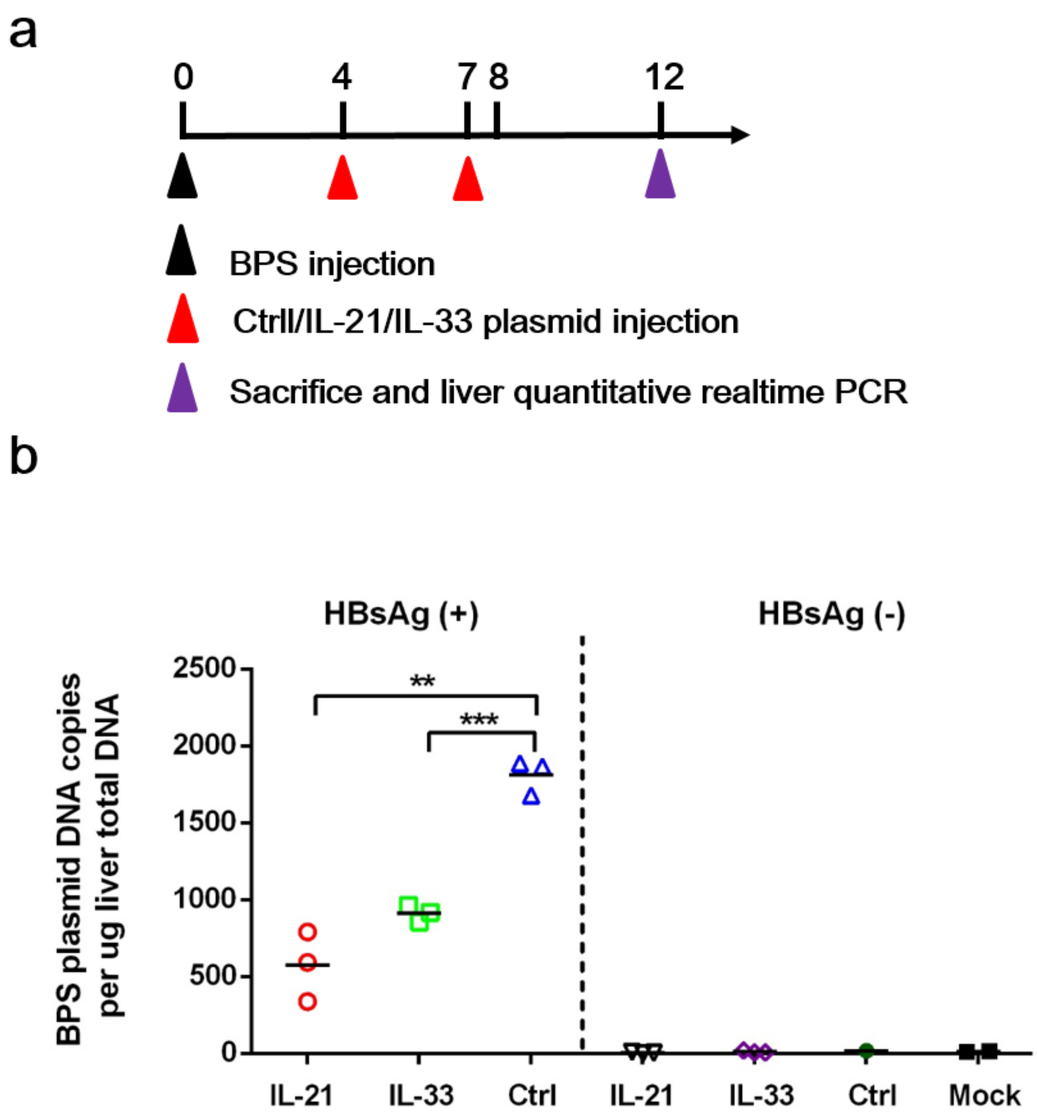

Supplementary Figure 31 Detection of HBV DNA in liver cell nuclei of BPS HDI mice post treatment with IL-21 and IL-33 plasmid injections. **a** Serum HBsAg positive BPS HDI BALB/c mice at 4 weeks post injection (w.p.i.) were injected hydrodynamically with IL-21 (n = 6) or IL-33 (n = 6) expression plasmid or control vector (n = 4) twice at 4 and 7 w.p.i. At 12 w.p.i., mice were sacrificed and serum and liver samples taken. **b** BPS plasmid DNA in liver cell nuclei from BPS HDI mice treated with injection of indicated plasmids was measured using quantitative realtime PCR. Three of 6 IL-21 treated mice, 3 of 6 IL-33 treated mice, and 1 of 4 control vector injected mice cleared serum HBsAg. Mice were grouped based on serum HBsAg positivity and group means are presented. Data are compared against control vector group and statistical significance calculated using unpaired two-tailed t-test. *p<0.05; ***p<0.001. Mock, naïve mice control (n = 2).

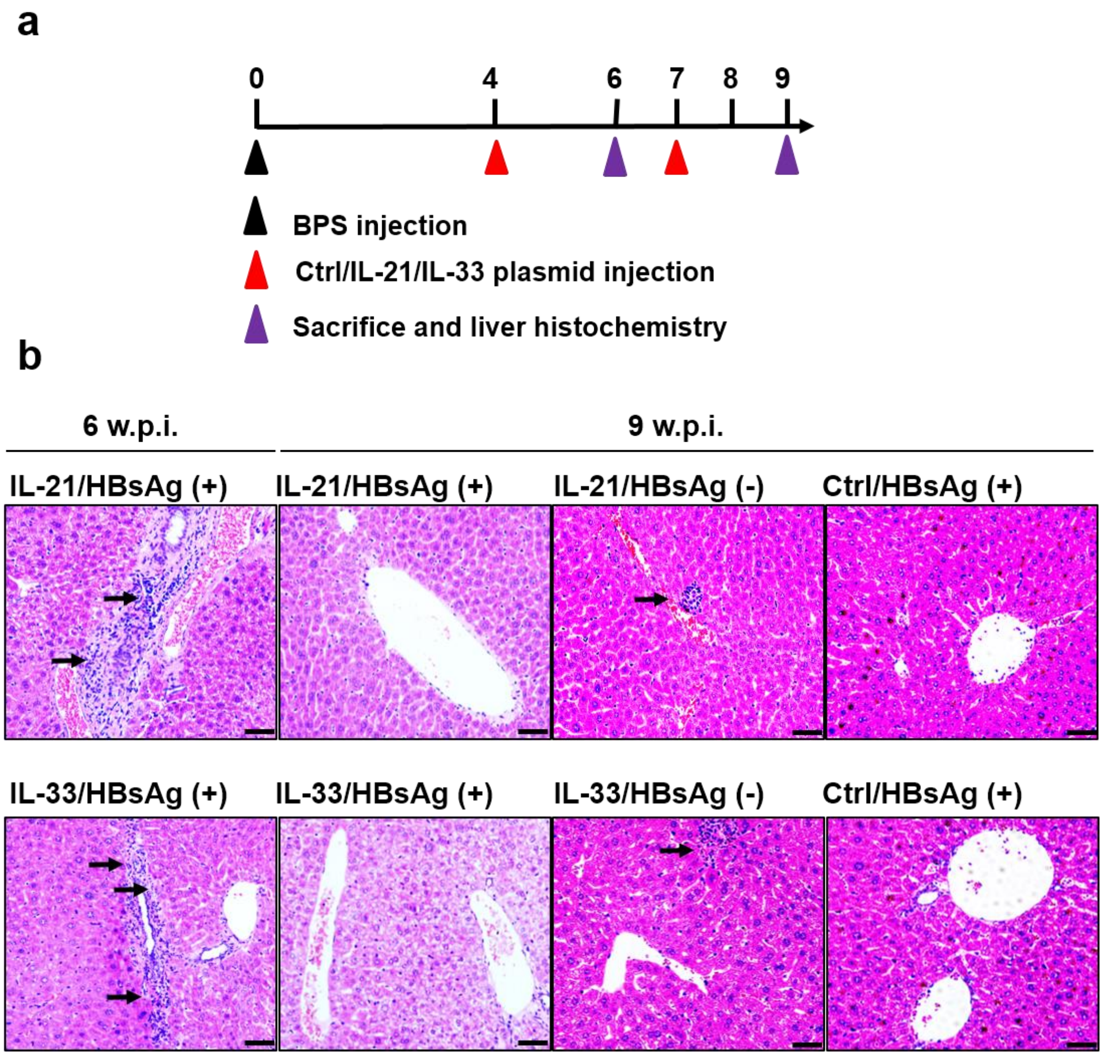

Supplementary Figure 32 Injection of IL-21 and IL-33 expression plasmid induces immune infiltration in liver of BPS HDI mice. **a** Serum HBsAg positive BPS HDI BALB/c mice at 4 weeks post injection (w.p.i.) were injected hydrodynamically with IL-21 or IL-33 expression plasmid or control vector twice at 4 and 7 w.p.i. At 6 w.p.i. and 9 w.p.i. (2 weeks post each treatment), randomly selected mice were sacrificed and serum and liver samples taken. **b** H&E staining of liver sections prepared in **a**. Time points when sections were prepared are shown. Treatment and serum HBsAg status at sacrifice are indicated at the top of each image. All treated mice were positive for serum HBsAg at 6 w.p.i. Representative images from at least 3 mice per group are shown. Arrows, infiltration foci. Scale bars, 50 µm.

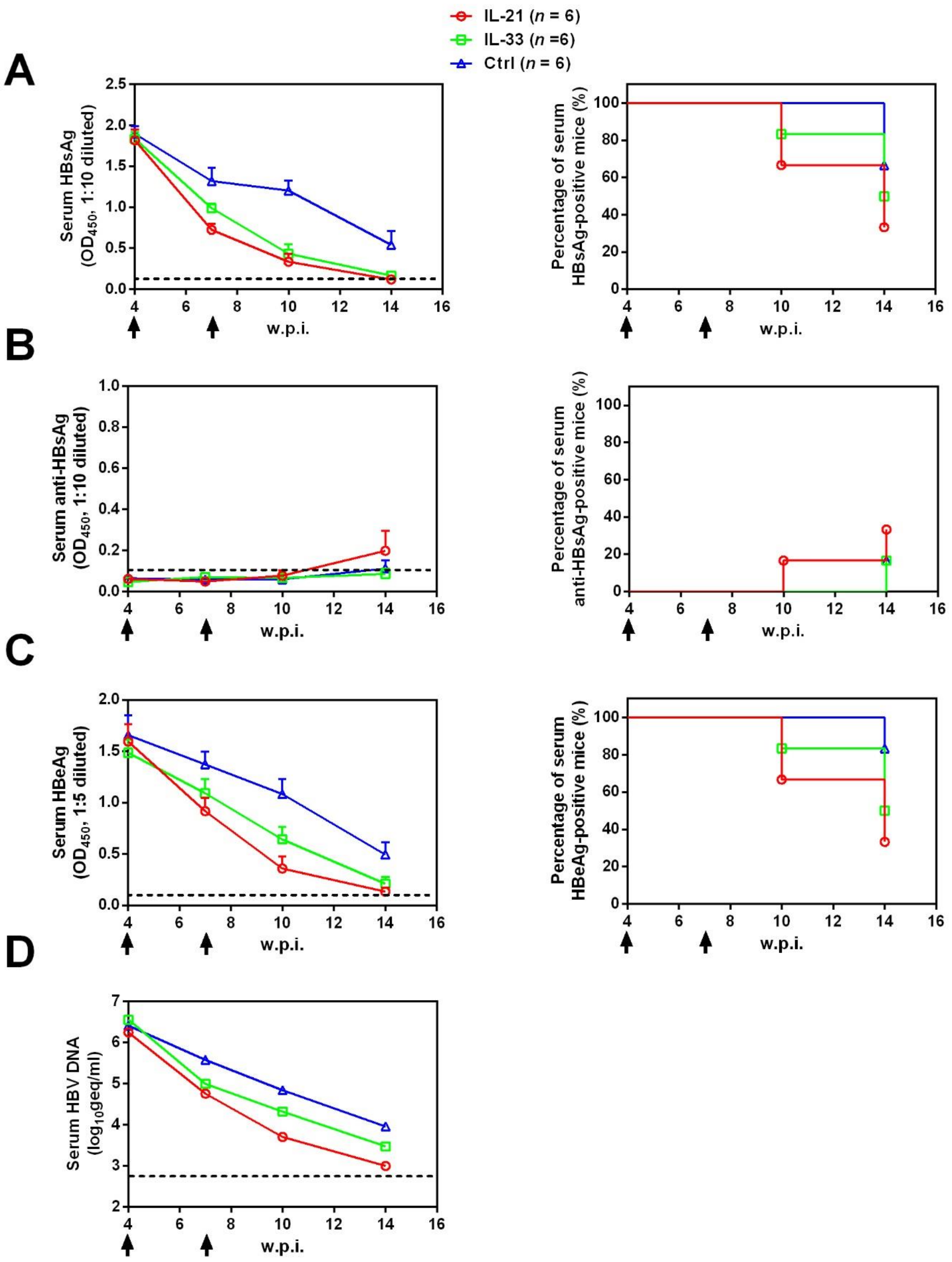

Supplementary Figure 33 Injection of IL-21 and IL-33 expression plasmid facilitated suppression and induce clearance of HBV in HBV persistence model dependent on AAV sequences. Four weeks after HBV replicon plasmid harboring AAV sequences were delivered via HDI into C57BL/6 mice, serum antigen positive mice were selected into groups (*n* = 6), and plasmids expressing murine IL-21 or IL-33, or vector controlwere hydrodynamically injected (arrows). Sera were collected at indicated time points and analysed for HBsAg **(a)**, HBsAb **(b)** and HBeAg **(c)** using ELISA. Group means and s.e.m. within group (left) and group positivity data (right) are presented. Pooled sera from each group were used for detecting HBV DNA using commercial quantitative assay **(d)**. Dotted lines represent cut-off thresholds (**a-c**) or detection limit (**d**). w.p.i., weeks post injection. geq, genome equivalent.

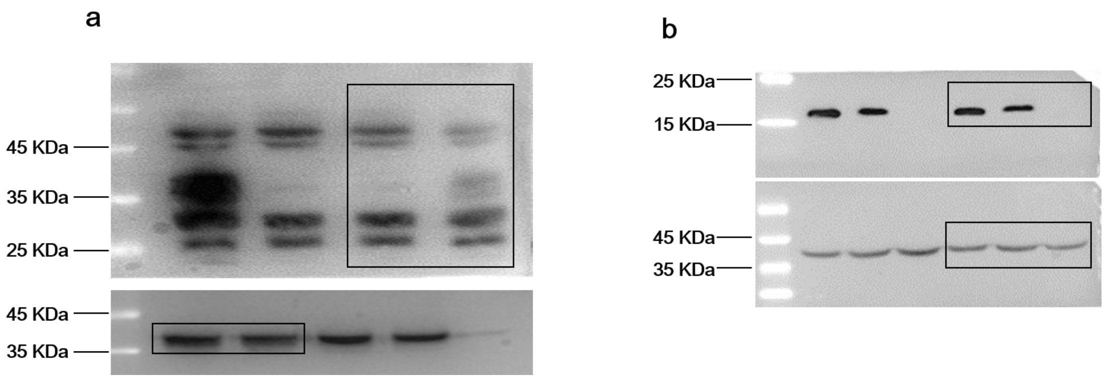

Supplementary Figure 34 Original uncropped Western blots of Supplementary Fig. 8a **(a)** and Supplementary Fig. 11a **(b)**. Rectangles indicate cropped parts used in respective figures.

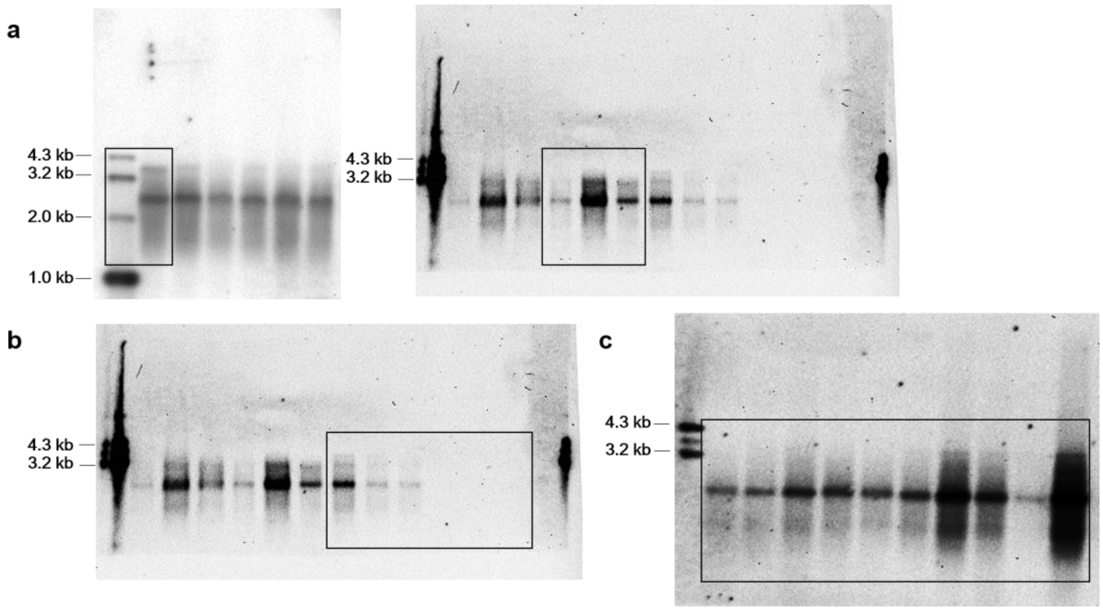

Supplementary Figure 35 Original uncropped Southern blots of Fig. 2c **(a)**, Fig. 3c **(b)**, and Supplementary Fig. 12 **(c)**. Rectangles indicate cropped parts used in respective figures.

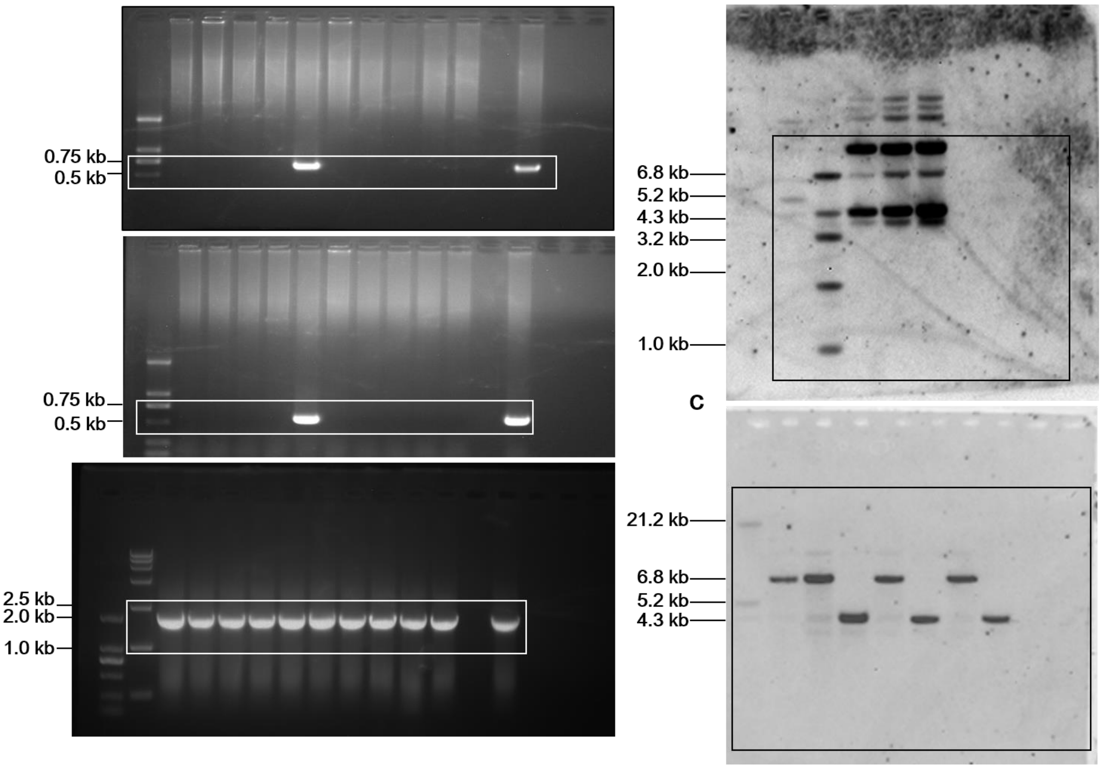

Supplementary Figure 36 Original uncropped agarose gel images and Southern blots of Supplementary Fig. 6. Rectangles indicate cropped parts used in the figure.

Supplementary Table 1. HBV strains used in this work.

Name	GenBank Accession Number	Genotype	Serotype	Source Patient Information					
				Gender	Age	HBsAg	HBeAg	HBV DNA (geq/ml)	ALT (U/L)
BPS	AF100309.1	B	adw	F	25	+	+		
B200	KR232337	B	adw	M	19	+	+	2.4E7	< 40
B6	KR152339	B	adw	F	34	+	+	7.1E7	126
A	AP007263.1	A	adw						
C*		C	adr						
D	V01460	D	ayw						
E	X75664.1	E	ayw						
H	AB516393.1	H	adw						

Available information is listed. *, the genotype C strain used in this work has not been submitted to GenBank, but has been used in multiple previous studies by us[1,2] and other groups. geq, genome equivalents.

Supplementary Table 2. Primer sequences

Target	Primer	Sequences
**Mouse *GAPDH***	Forward	CATGAGATAGCCTGGGGCTCACTACAGACC
	Reverse	GGAATGGAACCTTTAAAAGCAATGACGG
**HBsAg ORF**	Forward	ATGGAGAACATCGCATCAGGAC
	Reverse	TTAAATGTATACCCAAAGACAAA
**HBcAg ORF**	Forward	ATGGACATTGACCCGTATAAAG
	Reverse	CTAACATTGAGATTCCCGAGATT
**pUC18-BPS***	Forward	AGCGGATAACAATTTCACACAGGA (M13R)
	Reverse	GGGGTTGCGTCAGCAAACACTTGG (HBV 1176-1199)

* The targeted segment encompasses pUC18 vector and BPS HBV sequences, and annealing sites for the primers are listed in brackets.

Supplementary Table 3. Amino acid sequence differences between HBV ORFs encoded by BPS, B200 and B6.

ORF	Position	BPS	B200	B6
preC	Identical			
C	80	S	A	A
	95	I	L	L
X	5	L	L	V
	31	A	Q	P
	33	P	P	S
	39	P	T	P
	45	L	V	V
	47	T	A	T
	127	V	I	I
preS1	84	L	I	I
	85	L	F	L
	90	T	A	A
preS2	1	V	M	M
	32	V	L	L
	36	L	Q	Q
	41	A	T	A
S	44	G	G	E
	200	F	Y	Y
P	136	Y	H	H
	139	D	N	N
	163	T	A	T
	246	G	R	R
	264	P	H	H
	265	T	I	T
	270	N	S	S
	272	A	S	S
	300	R	H	H
	312	S	S	A
	319	P	S	P
	321	L	L	F
	331	S	T	S
	340	C	Y	C
	470	N	N	Y
	495	K	K	Q
	617	L	M	M
	720	L	V	V
	737	R	H	R
	805	F	L	L

Supplementary Table 4. Nucleotide sequence differences between BPS, B200 and B6 at promoter/enhancer locations.

**Element** (start - end)	**Position**	**BPS**	**B200**	**B6**
Cp (1643-1849)	1643	C	T	C
	1752	G	A	A
	1773	C	T	C
Xp (1235-1374)	1242	G	A	A
	1249	C	G	G
	1287	A	C	C
	1301	G	A	G
	1347	A	G	G
	1350	C	C	T
	1368	A	C	A
Sp1 (2219-2780)	2224	C	T	T
	2293	A	A	G
	2465	A	T	A
	2504	T	G	G
	2549	T	C	C
	2561	G	G	A
	2619	A	C	A
	2627	A	A	C
	2648	A	G	G
	2690	G	A	G
	2699	G	T	G
	2712	T	C	C
	2721	G	A	A
	2732	C	T	C
	2738	G	A	A
	2739	C	A	A
	2771	G	T	T
	2774	T	C	C
Sp2 (2809-3152)	3042	G	A	A
	3097	C	A	A
	3100	C	T	C
	3115	A	G	G
	3120	G	T	T
EnI (970-1240)	996	A	T	A
	1005	T	T	G
	1008	A	T	T
	1029	T	C	C
	1032	A	A	G
	1041	A	G	A
	1051	C	T	T
	1149	C	C	A
	1218	G	C	C
	1221	A	A	C
EnII (1685-1773)	1752	G	A	A
	1773	C	T	C

Supplementary References

1. Hong R, *et al.* Novel recombinant hepatitis B virus vectors efficiently deliver protein and RNA encoding genes into primary hepatocytes. *Journal of virology* **87**, 6615-6624 (2013).

2. Zhao X, *et al.* Quantitative Proteomic Analysis of Exosome Protein Content Changes Induced by Hepatitis B Virus in Huh-7 Cells Using SILAC Labeling and LC-MS/MS. *J Proteome Res* **13**, 5391-5402 (2014).
